# Promyelocytic leukemia protein (PML) controls breast cancer cell proliferation by modulating Forkhead transcription factors

**DOI:** 10.1002/1878-0261.12486

**Published:** 2019-05-16

**Authors:** Nikoleta Sachini, Panagiota Arampatzi, Antonios Klonizakis, Christoforos Nikolaou, Takis Makatounakis, Eric W.‐F. Lam, Androniki Kretsovali, Joseph Papamatheakis

**Affiliations:** ^1^ Department of Biology University of Crete Heraklion Greece; ^2^ Institute of Molecular Biology and Biotechnology Foundation for Research and Technology‐Hellas (FORTH) Heraklion Greece; ^3^ Department of Surgery and Cancer Imperial College London UK

**Keywords:** breast cancer, FOXO3‐FOXM1 network, growth arrest, PML, transcriptomics

## Abstract

The multitasking promyelocytic leukemia (PML) protein was originally recognized as a tumor‐suppressive factor, but more recent evidence has implicated PML in tumor cell prosurvival actions and poor patient prognosis in specific cancer settings. Here, we report that inducible PMLIV expression inhibits cell proliferation as well as self‐renewal and impairs cell cycle progression of breast cancer cell lines in a reversible manner. Transcriptomic profiling identified a large number of PML‐deregulated genes associated with various cell processes. Among them, cell cycle‐ and division‐related genes and their cognitive regulators are highly ranked. In this study, we focused on previously unknown PML targets, namely the Forkhead transcription factors. PML suppresses the Forkhead box subclass M1 (FOXM1) transcription factor at both the RNA and protein levels, along with many of its gene targets. We show that FOXM1 interacts with PMLIV primarily via its DNA‐binding domain and dynamically colocalizes in PML nuclear bodies. In parallel, PML modulates the activity of Forkhead box O3 (FOXO3), a factor opposing certain FOXM1 activities, to promote cell survival and stress resistance. Thus, PMLIV affects the balance of FOXO3 and FOXM1 transcriptional programs by acting on discrete gene subsets to favor both growth inhibition and survival. Interestingly, PMLIV‐specific knockdown mimicked ectopic expression *vis‐à‐vis* loss of proliferative ability and self‐renewal, but also led to loss of survival ability as shown by increased apoptosis. We propose that divergent or similar effects on cell physiology may be elicited by high or low PMLIV levels dictated by other concurrent genetic or epigenetic cancer cell states that may additionally account for its disparate effects in various cancer types.

**Abbreviations**
AKT: protein kinase B
DBD: DNA‐binding domain
FOX: Forkhead box
FOXM1: Forkhead box subclass M1
FOXO3: Forkhead box O3
PML‐NBs: PML nuclear bodies
PML: promyelocytic leukemia protein

## 1 Introduction

Breast cancer is one of the most prevalent causes of cancer‐related death in women worldwide. Breast tumors exhibit striking phenotypic and functional heterogeneity during the multistep course of tumorigenesis (Bertos and Park, 2011). In fact, this variability is mirrored in each of the ‘hallmarks of cancer’ defined by Weinberg and Hanahan namely sustaining proliferation signaling, mutational frequencies of oncogenes and tumor suppressors, invasiveness, metastatic potential, energy metabolism, immune destruction, and response to cytotoxic therapies (Hanahan and Weinberg, 2011). Extensive heterogeneity can be observed not only among different patients (intertumoral) but also within individual tumors (intratumoral) (Skibinski and Kuperwasser, 2015). Therefore, understanding the molecular networks governing breast cancer heterogeneity, including deregulated genes and their signaling pathways, is vital for improving the efficacy of existing agents and for developing novel strategies for personalized treatment.

The promyelocytic leukemia (*PML*) gene was first described at the chromosomal translocation breakpoint *t* (15;17) found in patients with acute promyelocytic leukemia (APL). This translocation generates a chimeric PML‐retinoic acid receptor‐a oncoprotein that blocks myeloid cell differentiation leading to leukemogenesis (de The *et al*., 1991). The human *PML* gene undergoes alternative splicing giving rise to seven main isoforms designated PMLI‐VII. PMLI to PMLVI contain a nuclear localization signal (NLS) and are predominantly localized in the nucleus, while PMLVII lacks the NLS and is cytoplasmic (Fagioli *et al*., 1992; Jensen *et al*., 2001; Nisole *et al*., 2013). All the PML isoforms share a common N‐terminal region containing the RBCC (RING‐finger, two B‐boxes (B1 and B2), and a α‐helical coiled‐coil domain) motif, while they differ in the central or in the C‐terminal region. The variable C termini of PML isoforms might provide different binding interfaces determining the different interacting partners and, thus, the functional specificity of each isoform (Nisole *et al*., 2013). PML protein is the core component of discrete subnuclear structures interspersed in the interchromatin space, termed PML nuclear bodies (PML‐NBs). PML‐NBs are dynamic, heterogeneous, proteinaceous particles found in most mammalian cell nuclei. They provide a microenvironment for protein sequestration, post‐translational modifications, and transcriptional regulation (Bernardi and Pandolfi, 2007). An ever‐growing number of proteins, including transcription factors/cofactors, ubiquitinases/deubiquitinases, kinases/phosphatases, sumo ligase/sumo1‐specific peptidases, and deacetylases, shuttle in and out PML‐NBs. Due to its different modes of function and its numerous interacting partners, PML and PML‐NBs are implicated in diverse cellular and physiological processes such as cellular senescence and apoptosis, genome maintenance and transcription, antiviral response, and stem cell maintenance, through distinct signaling pathways (Zhou and Bao, 2014; Hadjimichael *et al*., 2017; Hsu and Kao, 2018). For instance, PML is required for normal mammary gland development and lineage commitment of bi‐potent luminal progenitor cells, as *Pml* deletion in mice shifts the balance of luminal progenitors and impairs their terminal differentiation and gland size (Li *et al*., 2009).

The etiological involvement of PML in APL pathogenesis, its negative regulatory role on survival, and proliferation pathways (Salomoni *et al*., 2008) along with its downregulated expression in human cancers of multiple histological origins, including breast cancer (Gurrieri *et al*., 2004), provide strong evidence that PML acts as a tumor suppressor factor. Nevertheless, recent studies demonstrate that PML is upregulated in chronic myeloid leukemia (Ito *et al*., 2008) as well as in a subset of triple‐negative breast cancer (Carracedo *et al*., 2012). In the above cases, PML bears oncogenic properties via regulating cancer stem cell self‐renewal hence providing a selective prosurvival benefit to tumor cells.

Forkhead box (FOX) proteins constitute a superfamily of transcriptional regulators that all share an evolutionarily conserved Forkhead (FKH) or Winged helix DNA‐binding domain (DBD). These proteins fine‐tune a wide range of transcriptional programs crucial for embryonic development as well as homeostasis and repair of adult tissues (Lam *et al*., 2013). The family member, FOX subclass M1 (FOXM1), is highly expressed in various types of human malignancies, including breast cancer. FOXM1 is a master regulator of cell cycle progression and proliferation by orchestrating a network of G1/S and G2/M transitions, and M phase‐associated genes such as *CDC25B*, cyclin B (*CCNB1*), Aurora B kinase (*AURKA*), polo‐like kinase 1 (*PLK1*), survivin (*BIRC5*), and centromere protein A (*CENPA*), *CENPB*, and *CENPF*. Accordingly, loss of FOXM1 results in reduced DNA replication, mitotic spindle defects, and chromosome missegregation, and is homozygous lethal in mouse models (Laoukili *et al*., 2005; Wang *et al*., 2005; Wonsey and Follettie, 2005). Besides cell cycle progression, FOXM1 also controls genes implicated in angiogenesis, metastasis, epithelial–mesenchymal transition (EMT), stem cell expansion and renewal, senescence, and DNA repair. Thus, aberrant FOXM1 expression leads to tumor initiation, progression, metastasis, angiogenesis, and drug resistance (Bella *et al*., 2014). The oncogenic function of FOXM1 is partly counteracted by another member of the FOX family, Forkhead box O3 (FOXO3), which is a critical downstream effector of the phosphoinositide 3‐kinase‐protein kinase B (AKT) signaling pathway. FOXO3 transcriptional activity is tightly controlled by a complex combination of post‐translational modifications as well as protein–protein interactions that dictate its subcellular localization, protein expression levels, and DNA‐binding properties (Calnan and Brunet, 2008). FOXO3 directly binds to *FOXM1* promoter to repress its expression but also antagonizes FOXM1′s transcriptional output by competitively binding to the same target gene promoters (Lam *et al*., 2013). Although FOXO3 is generally reported to suppress cell proliferation and tumorigenesis, recent studies support that FOXO3 may also sustain tumor cell growth and induce drug resistance. Therefore, deregulation of the FOXO3‐FOXM1 axis can alter cell survival or proliferative state (Hornsveld *et al*., 2018; Yao *et al*., 2018).

In the present study, we show that PMLIV inhibits the growth of breast cancer cells by modulating FOXO3‐FOXM1 signaling. PMLIV overexpression represses the proliferation and self‐renewal of breast cancer cells and downregulates FOXM1 expression and protein activity. At the same time, PMLIV induction modulates FOXO3 activity and enhances its transcriptional program. Our results suggest that PMLIV orchestrates opposing actions on the FOXO3 and FOXM1 activity balance. We propose a model in which PMLIV represses breast cancer proliferation, but it may also promote long‐term survival and/or stress resistance by regulating FOXM1 and FOXO3 transcriptional programs.

## 2 Material and methods

### 2.1 Plasmids, DNA, and siRNA transfections

PMLIV and PMLIII expression vectors have been previously described (Gialitakis *et al*., 2010). For the co‐immunoprecipitation (IP) and colocalization studies, PMLIV and PMLIII were subcloned into dsRED monomer (Clontech, Mountain View, CA, USA). For the generation of stable cell line, the myc‐tagged PMLIV insert (described above) was first cloned into the bidirectional doxycycline (dox)‐inducible pBIG plasmid. Next, the region that carries the GFP‐tet‐PML was excised and reinserted into ClaI‐SalI backbone of the pLenti GFP PURO (Addgene #17448, Watertown, MA, USA) (Campeau *et al*., 2009) to generate the double GFP‐PML doxycycline‐inducible vector (toPMLxGFP). GFP‐NLS was constructed by cloning the SV40‐NLS sequence on pEGFP‐C (Clontech). GFP‐FOXM1 was generated by inserting the FOXM1 coding region from pCW57.1‐FOXM1c (Addgene #68810) (Barger *et al*., 2015) into pEGFP‐C (Clontech). The GST‐FOXM1 fragments used for the GST pull‐down assays have been previously described (Chen *et al*., 2013) and were generous gifts from A.D. Sharrocks (University of Manchester, Manchester, UK). The FOXO3 expression plasmid was gift from E. Lam and was fused into dsRED monomer plasmid (Clontech). Transient transfections were performed using the calcium phosphate DNA precipitation method or Lipofectamine 2000 (Thermo Fisher Scientific, Waltham, MA, USA) according to the manufacturer's instructions. The lentivirus production and infection protocol have been previously described in detail (Arampatzi *et al*., 2013).

For siRNA transfection, cells were seeded in six‐well plates and transfected with 50 pmol siRNA using the Lipofectamine 2000, according to the manufacturer's recommendations. The siPMLIV sequence that we used is as follows: 5′ UGAAAGUGGGUUCUCCUGG 3′ (Chen *et al*., 2018).

### 2.2 Cell culture and generation of stable cell lines

Human breast cancer cell lines: MDA‐MB‐231, T47D as well as HEK293T and Cos‐7 (obtained from ATTC, Manassas, VG, USA), were cultured in Dulbecco's modified Eagle's medium (DMEM), supplemented with 10% FBS and gentamycin in 5% CO_2_ and at 37 °C. Tumorspheres were grown in DMEM‐F12 1 : 1 (Gibco, Thermo Fisher Scientific) medium containing B27 (1 : 50), bFGF (20 ng·mL^−1^), epidermal growth factor (20 ng·mL^−1^), and 0.2% methylcellulose, in ultralow attachment plates. For the generation of doxycycline‐inducible cell lines, first an expressing pLenti CMV rtTA3 Blast (w756‐1) plasmid (Addgene #26429) was introduced into recipient cells that were selected by blasticidin. Next, the dox‐inducible toPMLxGFP was introduced.

For the generation of stable, knocked down cell lines, cells were infected by puromycin or G418 expressing lentiviral vectors carrying the relevant sh sequences, followed by drug selection. The shPML plasmid that we used was kindly provided by Everett *et al*. (2006). The sequence for shPMLIV is as follows: 5′ TGAAAGTGGGTTCTCCTGG 3′ (Chen *et al*., 2018). Subcloning of the specific shPMLIV into PLO.1 vector was done via AgeI and EcoRI sites flanking the 5′‐end of top and bottom shRNA oligo, respectively. Knockdown (KD) was evaluated by western and/or mRNA analysis using suitable primers.

### 2.3 Clonogenic assay

Two thousand cells were seeded into six‐well plates and incubated overnight. The next day, doxycycline was added in the medium to induce PMLIV expression. Cells were cultured for 10 days, and doxycycline was renewed in every change medium. Colonies were fixed with 4% PFA for 10 min at RT and then washed with PBS. Subsequently, colonies were stained with 0.5% crystal violet for 1 h, and then, plates were washed with tap water.

### 2.4 Cell cycle analysis

For cell cycle analysis, 100 000 cells from each sample were trypsinized, washed with PBS, treated with RNAse A for 30 min at 37 °C, and stained with propidium iodide (PI‐Sigma, Sigma‐Aldrich, St. Louis, MO, USA) according to the manufacturer's protocol. The analysis was conducted using a FACS Calibur analyzer (BD Biosciences, San Jose, CA, USA). The cell cycle profile was further analyzed using the modfit lt software (Verity Software House, Topsham, ME, USA).

### 2.5 RNA extraction and quantitative reverse transcription PCR (qRT‐PCR)

Total RNA was extracted using TRIzol reagent (Invitrogen, Carlsbad, CA, USA)/NucleoZOL (Macherey‐Nagel, Düren, Germany). Subsequently, 2 μg RNA was reversely transcribed to cDNA by M‐MuLV Reverse Transcriptase (NEB, Ipswich, MA, USA) supplemented with RNase inhibitor (NEB) according to the manufacturer's protocol. Relative abundance of each transcript was measured by quantitative real‐time PCR using SYBR Green I (Invitrogen). Relative mRNA expression was calculated after normalization against β‐actin levels. Primer sets used for real‐time qPCR analysis are listed in Table S1.

### 2.6 cDNA microarrays analysis

Total RNA was isolated from control and PMLIV OE cells using TRIzol (Invitrogen). The gene expression profile of cells was determined by GeneChip® Human Transcriptome Arrays 1.0, (Thermo Fisher Scientific) according to the manufacturer's instructions. The raw data were processed to extract the representative intensities from each probe set using affymetrix transcriptome analysis console software (Thermo Fisher Scientific). A threshold of fold change > 1.5 and *P* ≤ 0.05 was used to identify differentially expressed genes (DEGs) between the two conditions. Functional analysis was performed using the online tool g:profiler (Reimand *et al*., 2016) as well as rnea (Chouvardas *et al*., 2016).

### 2.7 Overlap analysis of gene lists

In order to determine whether PMLIV OE affects the same subset of genes that are differentially expressed upon FOXM1 KD in MDA‐MB‐231 cells, siFOXM1 datasets were retrieved from GEO (GSE25741, Platform ID: GPL6947). Using the GEO2R tool, a list of relative expression values was extracted for siFOXM1 versus siLuc (control). Subsequently, the same cut‐offs used for PMLIV OE analysis (fold change > 1.5 and *P*‐value ≤ 0.05) were applied to generate a list of DEGs for the FOXM1 KD dataset. The lists of PMLIV OE and FOXM1 KD DEGs were categorized into overexpressed and under‐expressed genes. The degree of overlap between PMLIV OE and FOXM1 KD DEGs was assessed by two Jaccard similarity indexes, one for each of the over‐ and under‐expressed gene lists. The Jaccard similarity index is defined as the intersection over the union of the two sets. The significance of the calculated Jaccard indexes was resolved by permutation analysis. For that reason, 10 000 randomized gene lists with the same size as the PMLIV OE DEG were generated using a custom Perl script. Successively, the 10 000 corresponding Jaccard indexes were calculated for overexpressed and under‐expressed genes. After computing the mean and standard deviation of the Jaccard indices of the permutated lists, the initial, calculated Jaccard index was compared to the mean of this distribution based on *z*‐score.

The same approach was used for the overlap analysis of PMLIV OE and constructively activated FOXO3 DEGs. The FOXO3 datasets were retrieved from GEO (GSE113479, Platform ID: GPL24915). The statistical significance of the triple overlap sets (PMLIV OE, FOXM1 KD, FOXO3 activation) was assessed using the R package ‘SuperExactTest’ (Wang *et al*., 2015) which calculates the statistical distributions of multiset intersections based on combinatorial theory.

### 2.8 Western blot analysis

Whole cell lysates were prepared using RIPA cell lysis buffer (25 mm Tris pH 7.6, 150 mm NaCl, 1% NP‐40, 1% deoxycholate, 0.1% SDS, 1 mm PMSF) containing protease inhibitor cocktail (Complete; Sigma), and protein concentration was determined by Bradford assay. Equal amounts of cell lysates were subjected to SDS/PAGE, followed by immunoblotting. The antibodies used in this study are listed below: β‐actin (sc‐47778; Santa Cruz, Santa Cruz, CA, USA), PML (sc‐5621; Santa Cruz), PML (sc‐377340; Santa Cruz), ppRB (9309; Cell Signaling, Danvers, MA, USA), p‐pRB (9308; Cell Signaling), p53 (sc‐6243; Santa Cruz), p21 (sc‐397; Santa Cruz), FOXM1 (sc‐502; Santa Cruz), FOXM1 (sc‐376471; Santa Cruz), TBP (ab28175), GFP (9996; Cell Signaling), RCFP (anti‐KillerRed; AB961; Evrogen, Moscow, Russia), CCNB1 (sc‐752; Santa Cruz), PLK1 (4513; Cell Signaling), CCND1 (sc‐8396; Santa Cruz), AURKA (3092; Cell Signaling), RAD51 (sc‐8349; Santa Cruz), β‐tubulin (2146; Cell Signaling), FOXO3 (2497; Cell Signaling), and FOXO3 (ab12162; Abcam, Cambridge, UK).

### 2.9 Measure of FOXM1 protein turnover

The turnover rate of endogenous FOXM1 in MDA‐MB‐231 was determined using cycloheximide (CHX; Sigma) for protein synthesis inhibition. PMLIV was induced for 12 h, and then, CHX was added to the culture media to a final concentration of 100 μg·mL^−1^. Cells were harvested at the indicated time points, and equal amounts of cell lysates were subjected to SDS/PAGE and analyzed by immunoblotting.

### 2.10 Protein immunoprecipitation

Immunoprecipitation of *in vivo* interacting protein complexes was performed using the MDA‐MB‐231 PMLIV OE cell line or HEK293T cell extracts prepared by RIPA cell lysis buffer as described above. Two hundred microgram of protein extracts was incubated with primary antibody overnight at 4 °C. The following day, 20 μL of protein G beads was added to each sample after washing with IP buffer (25 mm Tris/HCl pH 7.6, 150 mm NaCl), and reactions were incubated at 4 °C for three additional hours. Nonspecific proteins were washed away three times with NETN buffer (10 mm Tris/HCl pH 8.0, 250 mm NaCl, 5 mm EDTA, 0.5% NP‐40, 1 mm PMSF). SDS sample buffer was added, and the samples were boiled prior to SDS/PAGE analysis. Input lanes represent 10% of the lysate used for the IP.

### 2.11 GST pull‐down assay

GST‐FOXM1 fusion constructs were expressed in BL21‐Star™ (DE3) pLysS cells (Thermo Fisher Scientific), and crude bacterial lysates were prepared by sonication in cold lysis buffer (50 mm Tris/HCl pH 8.0, 100 mm NaCl, 1 mm EDTA, pH 8.0, 2% NP‐40, 1 mm DTT, 1 mm PMSF). To test the interaction between FOXM1 and PMLIV, GST‐fusion proteins were freshly purified by glutathione‐Sepharose beads (GE Healthcare, Chicago, IL, USA), washed two times with lysis buffer and one time with GST‐Wash buffer (300 mm KCl, 20 mm HEPES pH 7.9, 0.1% NP40, 5 mm MgCl_2_), and resuspended in 200 μL GST‐interaction buffer (150 mm KCl, 20 mm HEPES pH 7.9, 0.1% NP40, 5 mm MgCl_2_) and mixed with 200 μg of HEK‐293T cell lysate overexpressing (OE) mRED‐PMLIV. The binding reaction was incubated for 3 h at 4 °C. Beads were washed three times with GST‐Wash buffer (600 mm KCl, 20 mm HEPES pH 7.9, 0.1% NP40, 5 mm MgCl_2_) and resuspended in SDS sample buffer. Samples were subjected to SDS/PAGE and analyzed by immunoblotting.

### 2.12 ChIP assay

A total of 3 × 10^7^ MDA‐MB‐231 cells were fixed in 1% formaldehyde for 10 min at room temperature. Formaldehyde was subsequently quenched with 0.125 m glycine for 5 min, and the cells were washed twice with ice‐cold PBS. Cells were lysed in lysis buffer [50 mm HEPES (pH 7.5), 140 mm NaCl, 1 mm EDTA, 10% glycerol, 0.5% NP‐40, 0.25% Triton X‐100, and 1 mm PMSF] on ice for 20 min. Cells were pelleted, resuspended in lysis buffer, pass through 0.5‐mL syringe, and centrifuged for three successive times. After the last centrifugation, pellet was resuspended in 2 mL shearing buffer [0.05 m Tris (pH 8.0), 0.3% SDS, 0.01 m EDTA, and 1 mm PMSF] for 30 × 10^6^ initial cell number and sonicated to an average length of 500 bp, as verified by agarose gel electrophoresis. IP was performed with the equivalent of 30 × 106 cells per sample diluted five times with ChIP dilution buffer (10 mm Tris/HCl pH 8.0, 0.01 m EDTA, 100 mm NaCl, 0.01% SDS, 1% Triton X‐100, 1 mm PMSF) to which 2–10 μg of each antibody was added and rotation followed at 4 °C overnight. The antibody–chromatin reactions were precipitated with BSA preblocked protein G agarose beads for 3 h by rotation. Unbound chromatin was removed by three washes in RIPA buffer (50 mm HEPES‐KOH pH 7.5, 500 mm LiCL, 1 mm EDTA pH 8, 1% NP‐40, 0.7% Na‐deoxycholate) once with TE, pH 8.0. Immunoprecipitated chromatin was reverse cross‐linked in 1% SDS, 50 mm Tris/HCl pH 8.0, 10 mm EDTA pH 8.0 by overnight incubation at 65 °C. Following the reverse cross‐linking, samples were treated with 100 mg·mL^−1^ RNAse A and subsequently with 20 mg·mL^−1^ proteinase K at 55 °C for 3 h. DNA was purified with phenol/chloroform extraction and precipitated with ethanol and glycogen precipitated. Enrichment of specific sequences in the immunoprecipitated DNA was measured by real‐time PCR with SYBR green I and expressed as a percentage of input DNA. The primer sets that were used are provided in Table S1.

### 2.13 Immunofluorescence and live microscopy

Cells for immunostaining were cultured on glass coverslips and fixed in 4% PFA/1× PBS for 12 min at room temperature, permeabilized with 0.5% Triton X‐100/1× PBS for 15 min, and rinsed repeatedly with 1× PBS. Subsequently, samples were blocked with 1% BSA/1× PBS for 1 h and then were incubated with primary antibodies overnight/1 h. Following washes with PBS, secondary antibody was added to the samples for an hour. Secondary antibody was washed again three times with PBS, and cells were then counterstained with DAPI (Sigma) and mounted on microscope slides. Samples were analyzed with a Zeiss (Oberkochen, Germany) Axioscope 2 Plus microscope equipped with a Bio‐Rad (Hercules, CA, USA) Radiance 2100 laser scanning system and Lasersharp 2000 imaging software.

Cells for live microscopy were grown in Lab‐Tech chambers (Thermo Fisher Scientific), were transfected as mentioned above, and 48 h post‐transfection were examined within 1 h at room temperature with a Zeiss Axioscope 2 Plus microscope. Fluorescence recovery after photobleaching (FRAP) analysis was done using a standard region of interest (ROI) and monitoring close to saturation of recovery. After subtracting the background, fluorescent intensities were normalized against a companion unbleached ROI in the same cell.

### 2.14 Statistical analysis

Statistics were determined using the xlstat software (Addinsoft Inc, Long Island City, NY, USA). Values were presented as the mean + SD.

## 3 Results

### 3.1 PMLIV suppresses the proliferation and self‐renewal of triple‐negative breast cancer cells

**Figure 1:**
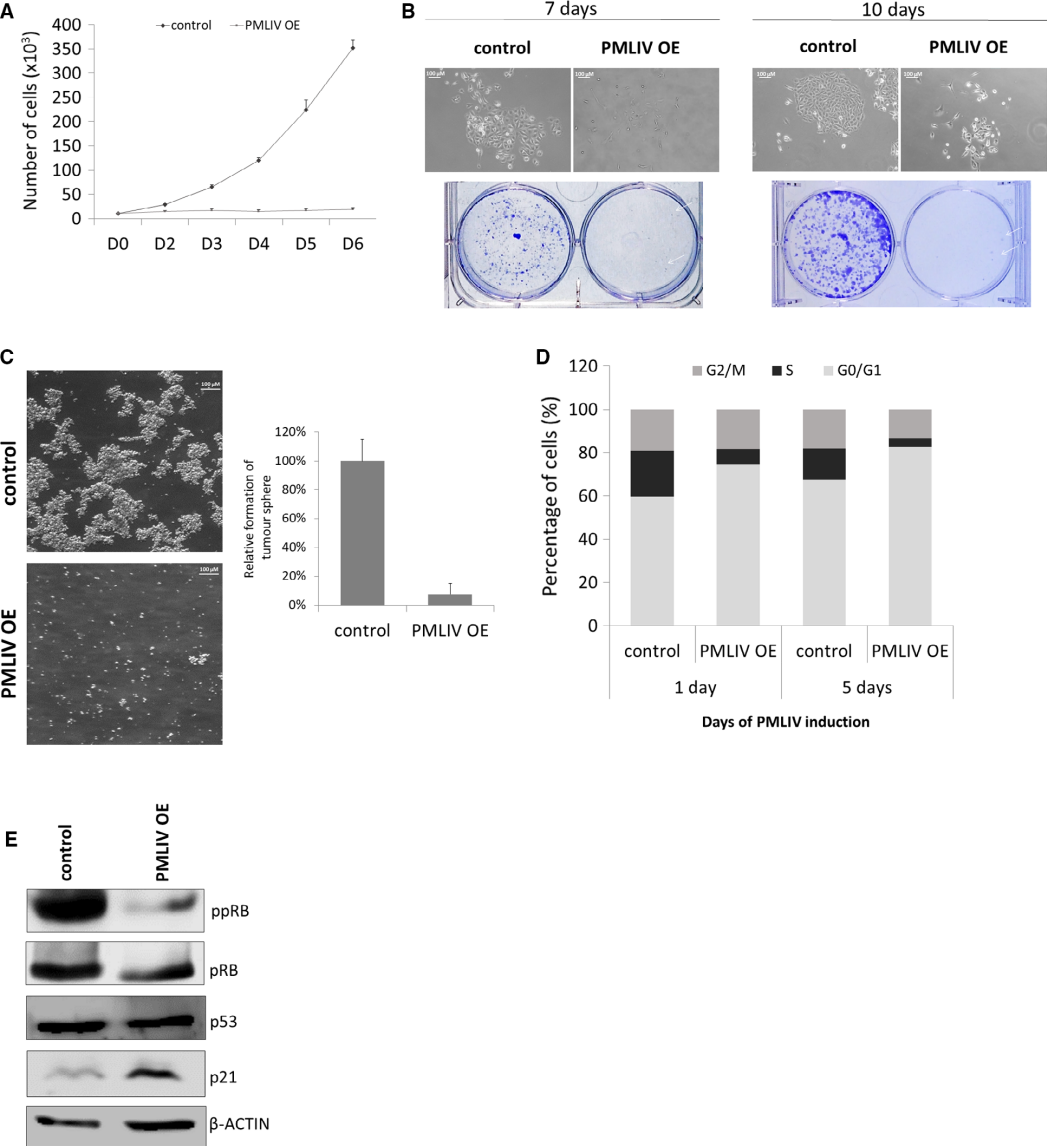
PMLIV induction results in growth and cell cycle arrest of breast cancer cells. (A) Cell growth of control and PMLIV OE MDA‐MB‐231 cells. Data represent the mean + SD of three independent experiments (*n* = 3). (B) Clonogenic assays of MDA‐MB‐231 cells evaluated at day 10 post‐PMLIV induction. White arrows indicate colonies of PMLIV OE cells (scale bar, 100 μμ). (C) Tumorsphere formation of control and PMLIV OE MDA‐MB‐231 cells. For the tumorsphere‐forming assay, 1000 cells·mL^−1^ were seeded in triplicate. After 8 days in culture, tumorspheres were counted using a light microscope. Results are presented as mean + SD of three independent experiments (*n* = 3; scale bar, 100 μμ). (D) Cell cycle analysis of control and PMLIV OE cells stained with PI and analyzed using flow cytometry assayed at days 1 and 5 post‐PML induction. (E) Western blot analysis of control and PMLIV OE cells for cell cycle regulators. β‐actin was used as a loading control.

To study the role of PML in breast cancer, we first examined the expression levels of PML in breast cancer cell lines of different molecular subtypes (Fig. S1A). In agreement with previous reports, PML expression varies among breast cancer cell lines, as well as among breast cancer patients’ samples even of the same molecular subtype (Carracedo *et al*., 2012; Martin‐Martin *et al*., 2016; Ponente *et al*., 2017). TCGA‐METABRIC breast patient datasets show a wide variation of PML mRNA (Curtis *et al*., 2012; Pereira *et al*., 2016) (http://www.cbioportal.org). To investigate the molecular mechanisms underlying the involvement of PML in cell proliferation and self‐renewal pathways in breast cancer, we used the PMLIV isoform since it is associated with apoptosis, senescence, and DNA damage (Nisole *et al*., 2013). Thus, a transgene expressing PMLIV upon doxycycline treatment was stably integrated into MDA‐MB‐231 cells, a claudin‐low, triple‐negative, aggressive breast cancer cell line, tο generate an inducible PMLIV OE system. To assess the efficiency of PML induction in the stable cell line, we measured its endogenous, expression levels before (control) and after doxycycline treatment (PMLIV OE). PMLIV mRNA and protein levels were markedly increased when doxycycline was added to the cells. Moreover, immunostaining for PML revealed an intense PML‐NBs morphology and size difference between PMLIV OE and control cells (Fig. S1B). The cell proliferation rate of PMLIV OE cells decreased compared to the control cells, indicating that PMLIV practically arrests breast cancer growth *in vitro* (Fig. 1A). The antiproliferative function of PMLIV was also confirmed by clonogenicity assay, since PMLIV induction resulted in dramatically fewer and smaller colonies, suggesting that PMLIV represses colony formation of MDA‐MB‐231 cells (Fig. 1B). We also examined whether PMLIV affects the self‐renewal capacity of MDA‐MB‐231 cells growing as 3D tumorspheres. PMLIV overexpression significantly decreased sphere formation efficiency of MDA‐MB‐231 cells, implying that PML inhibits the self‐renewal of breast cancer stem cells (Fig. 1C). Based on the inhibitory effects of PML overexpression on cell growth rate, clonogenicity, and self‐renewal capacity, we next assessed the impact of PMLIV on cell cycle progression after 1 and 5 days of PMLIV induction. At day 1 of PMLIV OE, there was an increase in cell fractions in the G1 phase with a concomitant decrease of cells in S phase. The effect was even more pronounced after 5 days of PMLIV OE, with a dramatic reduction of S phase cells accompanied by a slight decrease in cells in G2/M (Figs 1D and S1C). Furthermore, PMLIV OE cells were characterized by reduced levels of ppRB as expected, while total pRB and p53 levels did not show significant change (Fig. 1E).

We also examined the effect of PMLIV overexpression in another breast cancer cell line with luminal characteristics, T47D. The efficiency of PMLIV induction was assessed at mRNA and protein level by qPCR and western blot, while the formation of PML‐NBs, with immunofluorescence (Fig. S1D). Similarly, to PMLIV OE in MDA‐MB‐231 cells, PMLIV OE in T47D cells drastically inhibited proliferation in conventional media but partly inhibited sphere‐forming ability. In addition, PMLIV induction also resulted in increased G1 phase population followed by a decrease in cells in S and G2/M phases (Fig. S1E).

Taken together, our findings suggest that PMLIV induction represses breast cancer cell cycle progression and proliferation in both cases but affects sphere formation in the luminal type T47D much less effectively than in MDA‐MB‐231 cells.

### 3.2 PMLIV‐elicited gene expression profile of MDA‐MB‐231 cells

**Figure 2:**
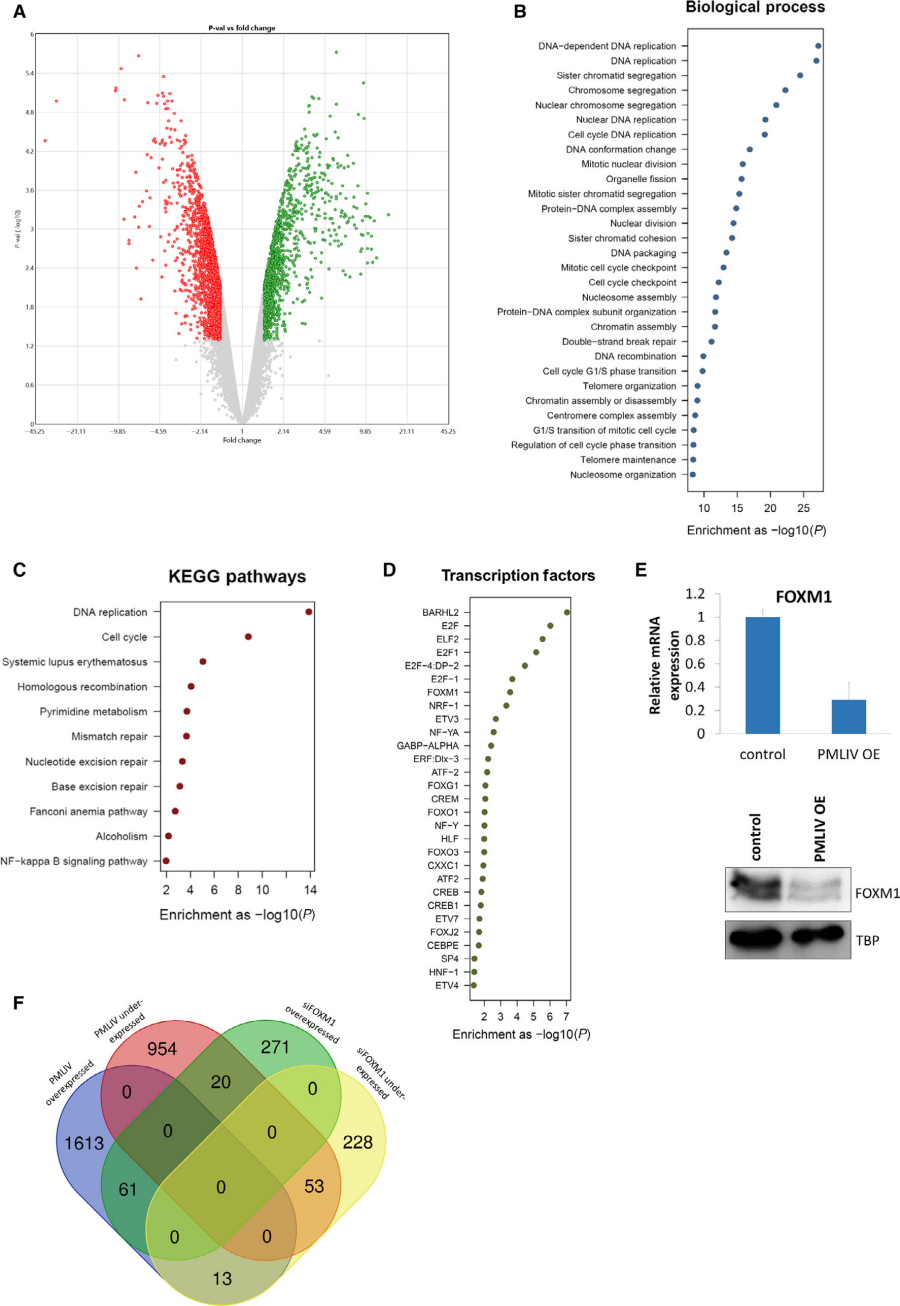
Changes of global gene expression upon PMLIV induction in MDA‐MB‐231 cells. (A) Volcano plot depicting the distribution of DEGs after PMLIV OE (fold change > 1.5, *P* ≤ 0.05). (B, C) Functional enrichment analysis of the DEGs upon PMLIV induction performed using g:profiler. Scatter plots showing significantly enriched GOs and KEGG pathways, respectively. (D) Scatter plot illustrating the significantly enriched transcription factors when PMLIV is overexpressed. (E) FOXM1 relative mRNA expression levels and protein levels upon PMLIV induction. Error bars indicate SD from three independent experiments (*n* = 3). (F) Venn diagram depicting the overlap between PMLIV and FOXM1 targets in MDA‐MB‐231 cells. FOXM1 KD datasets were retrieved from GEO (GSE25741).

To further dissect PML's role in breast cancer growth, we performed genome‐wide expression analysis on PMLIV OE and control cells (GSE119583). Using a *P*‐value ≤ 0.05 and fold change cut‐off of 1.5, transcriptomic profiling revealed that 2714 genes were differentially expressed when PMLIV is induced, with 1687 being overexpressed and 1027 under‐expressed (Fig. 2A). Selected genes from both groups implicated in different biological processes (cell cycle, DNA replication and repair, chromatin modification, and immune response) were validated by qPCR (Fig. S2A). As expected, PML expression was upregulated in our dataset, validating our experimental design. Immune‐ and inflammation‐related genes, such as *IL23R*,* HLA*,* TNFRSF9*, were also significantly upregulated upon PMLIV induction in agreement with the well‐established PML's role in inflammatory responses and antiviral function (Hsu and Kao, 2018). The complete list of PML‐deregulated genes is provided in the Table S2.

Gene ontology (GO) and KEGG pathway functional enrichment analysis using the g:profiler software (Reimand *et al*., 2016) demonstrated that cell cycle‐ and especially mitotic‐related biological processes and pathways were over‐represented when PMLIV is overexpressed corroborating with the cell cycle arrest phenotype that we observed upon PMLIV induction and the known antiproliferative function of PML reported in the literature (Fig. 2B,C). In fact, genes involved in the cell cycle phase progression and transitions (e.g., *CDKs, CCNBs, PLKs, AURKA/B*), spindle organization (*SPAG5, ASPM*), cytokinesis (*KIF protein members*), and chromosome segregation (*TOP2A, BRCA1, CENPE, CENPF*) were under‐expressed after PMLIV forced expression. We also performed functional analysis using the Regulatory Network Enrichment Analysis (rnea) software (Chouvardas *et al*., 2016). The data obtained from rnea were in agreement with the previous analysis, but they provided us further insight into the GO categories and KEGG pathways that were significantly enriched either in overexpressed genes or in under‐expressed genes. Importantly, overexpressed genes upon PMLIV induction are over‐represented in immune‐ and inflammation‐related GO terms and KEGG pathways, whereas DNA and RNA processes associated GO categories and pathways are enriched in under‐expressed genes (Fig. S2B).

In addition, we evaluated the effect of PMLIV induction on MDA‐MB‐231 cultured passage 1 spheres by microarrays. Results showed that the PMLIV OE deregulated genes in sphere conditions highly resemble those of the cells growing in monolayer. Again, mitosis‐, cell cycle‐, and DNA process‐related GOs and pathways were significantly enriched in under‐expressed genes. As expected, each condition presented unique GO categories and pathways over‐represented mainly by overexpressed genes (Fig. 2SC).

The functional analysis further revealed the enrichment of transcription factors that regulate cell proliferation such as E2F, NFY, CREB, and members of the FKH transcription factor family (Fig. 2D). Among them, FOXM1 was itself suppressed in both mRNA and protein levels after PMLIV forced expression (Fig. 2E). Likewise, FOXM1 mRNA and protein levels as well as FOXM1′s downstream targets were decreased in PMLIV OE T47D cells (Fig. S2D).

Of note, comparison of our data with published genome‐wide expression data of FOXM1 KD in MDA‐MB‐231 cells (Park *et al*., 2012) showed PMLIV OE DEGs to significantly overlap with FOXM1 targets (Fig. 2F), implying a functional regulatory relation between them. Sixty‐one overexpressed and 53 under‐expressed genes were common in the PMLIV OE and siFOXM1 datasets. Interestingly, the common under‐expressed genes of PMLIV OE and FOXM1 KD were cell cycle‐related, whereas the common overexpressed genes were immune system process‐related (Fig. 2SE). This analysis shows that PMLIV has a bias in opposing both FOXM1 positively and negatively regulated genes, since enrichment for common genes between PMLIV OE under‐expressed and siFOXM1 overexpressed datasets and *vice versa* was not statistically significant.

Collectively, the transcriptomic profiling of MDA‐MB‐231 cells revealed that multiple transcriptional and epigenetic networks are altered upon PMLIV induction. FOXM1 transcription factor is a prominent PML target since it is downregulated when PMLIV is overexpressed and is enriched in the gene set functional analysis.

### 3.3 FOXM1 interacts with PMLIV and colocalizes in PML‐NBs

**Figure 3:**
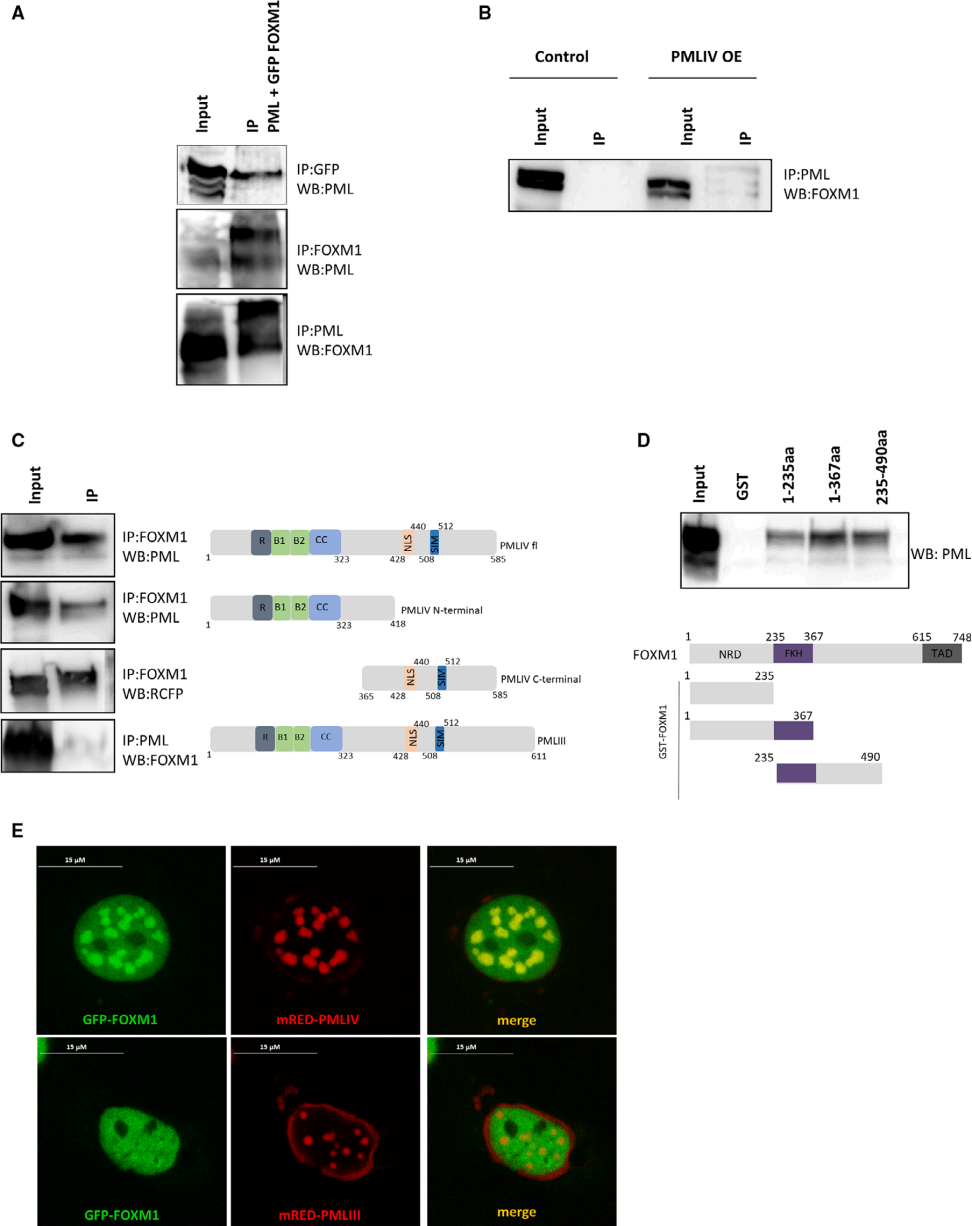
FOXM1 interacts with PMLIV. (A) Co‐IP was performed with a GFP, FOXM1, or PML antibody on lysates from transfected HEK293T cells. Inputs (1/10 of IP) and immunoprecipitates were blotted and probed for PML or FOXM1. (B) Co‐IP of PML and FOXM1 on lysates of MDA‐MB‐231 PMLIV OE cells. (C) Co‐IP of PML and FOXM1 on lysates from transfected HEK293T cells with the indicated constructs. (D) GST pull‐down assay using the indicated GST‐FOXM1 fusion proteins and total cell extracts from PMLIV‐transfected HEK293T cells. Interacting PMLIV revealed by western blotting. (E) Subnuclear localization of GFP‐FOXM1 and mRED‐PMLIV in Cos‐7 cells (scale bar, 15 μM).

To explore the mechanism by which PMLIV represses FOXM1 expression, we examined PML‐FOXM1 association *in vivo* by co‐IP experiments. Cell lysates from HEK293T cells transiently cotransfected with GFP‐FOXM1 and mRED‐PMLIV were used for IPs to test whether FOXM1 interacts with PMLIV. The results showed that FOXM1 co‐immunoprecipitated with PMLIV and *vice versa*, suggesting that PMLIV and FOXM1 physically interact when both proteins are ectopically expressed (Fig. 3A). To further confirm this, we performed co‐IP assays on the endogenous FOXM1 protein in MDA‐MB‐231 PMLIV OE cells. Consistently, FOXM1 co‐immunoprecipitated with PML affirming that FOXM1 interacts with PML (Fig. 3B).

All known human PML isoforms share an identical N‐terminal region, which contains a RBCC/TRIM motif responsible for homo‐multimerization and the formation of macromolecular complexes. The variations in C termini provide different docking sites for PML's interacting partners and mediate its functional specificity (Nisole *et al*., 2013). To examine whether the FOXM1‐PML interplay is isoform‐specific and which PML domain is responsible for this association, we transiently cotransfected HEK293T cells with GFP‐FOXM1 and either PMLIV full‐length or PMLIV N‐terminal or PMLIV C‐terminal deletions of PMLIV or the PMLIII that carries a different carboxyl‐terminal region. For comparison, we used the PMLIII isoform that is not linked to cell proliferation but rather to centrosome duplication (Xu *et al*., 2005), genome stability (Wu *et al*., 2009), and resistance to viruses (Chelbi‐Alix *et al*., 1998; Regad *et al*., 2001; Pampin *et al*., 2006). The results demonstrated that FOXM1 interacted strongly with PMLIV full‐length and with PMLIV C‐end, weaker with PMLIV N‐end, and hardly with PMLIII (Fig. 3C). These data suggest that FOXM1 specifically interacts with PMLIV and that FOXM1 could be one of the main PML's interacting partners accounting for the PMLIV isoform specificity of antiproliferative function. Having confirmed that PMLIV physically associates with FOXM1, we next examined whether they interact directly *in vitro* using GST pull‐down assays. The quality and quantity of GST‐FOXM1 fusion proteins were both checked with Coomassie brilliant blue staining (Fig. S3A). PMLIV bound to FOXM1 1–367aa which contains the DBD of FOXM1 with stronger affinity compared to the FOXM1 1–235aa and the 235–490aa, which correspond to the negative regulatory domain and part of DBD, respectively (Fig. 3D).

Promyelocytic leukemia‐NBs act as storage or catalytic platforms for numerous proteins providing a microenvironment for potential mutual regulation between PML and its interacting components. In consequence, FOXM1 could be regulated by PML in PML‐NBs. To test whether FOXM1 colocalizes in PML‐NBs, we cotransfected Cos‐7 cells with GFP‐FOXM1 and mRED‐PMLIV and we examined their distribution using confocal microscopy. GFP‐FOXM1 displayed diffused nuclear pattern but was also localized in distinct foci, while PMLIV‐mRED formed discrete nuclear speckles typical of PML‐NBs. The overlay of the images demonstrated that FOXM1 colocalizes in PML‐NBs (Fig. 3E). GFP‐NLS and GFP‐FOXM1 were used as controls (Fig. S3B). Our previous co‐IP assays suggest that PML‐FOXM1 interaction is isoform‐specific, as FOXM1 strongly associates with PMLIV and scarcely with PMLIII. These results were further supported by the colocalization experiments as we noticed that GFP‐FOXM1 did not colocalized with PML‐NBs formed by PMLIII mRED overexpression (Fig. 3E). To examine the dynamics of the observed PML‐FOXM1 association, we carried out FRAP experiments. FOXM1 showed much faster recovery compared to the structural nuclear body components PMLIV and PMLIII, indicating that their interaction is dynamic and that FOXM1 is one of PML‐NBs cargo proteins and not a constitutive component of PML‐NBs (Fig. S3C).

Taken together, the above findings show that FOXM1 interacts with specific PML isoforms and that the interaction is primarily mediated via PML's C‐terminal and FOXM1′s DBD domains, respectively. Moreover, FOXM1 colocalizes in PML‐NBs. Therefore, PML‐NBs could directly recruit and modulate FOXM1 activity and/or affect its expression. Since FOXM1 is an auto‐regulatory protein, it is most likely that PML interferes with both its expression and its function.

### 3.4 PMLIV induction downregulates FOXM1 expression and its recruitment to target genes

**Figure 4:**
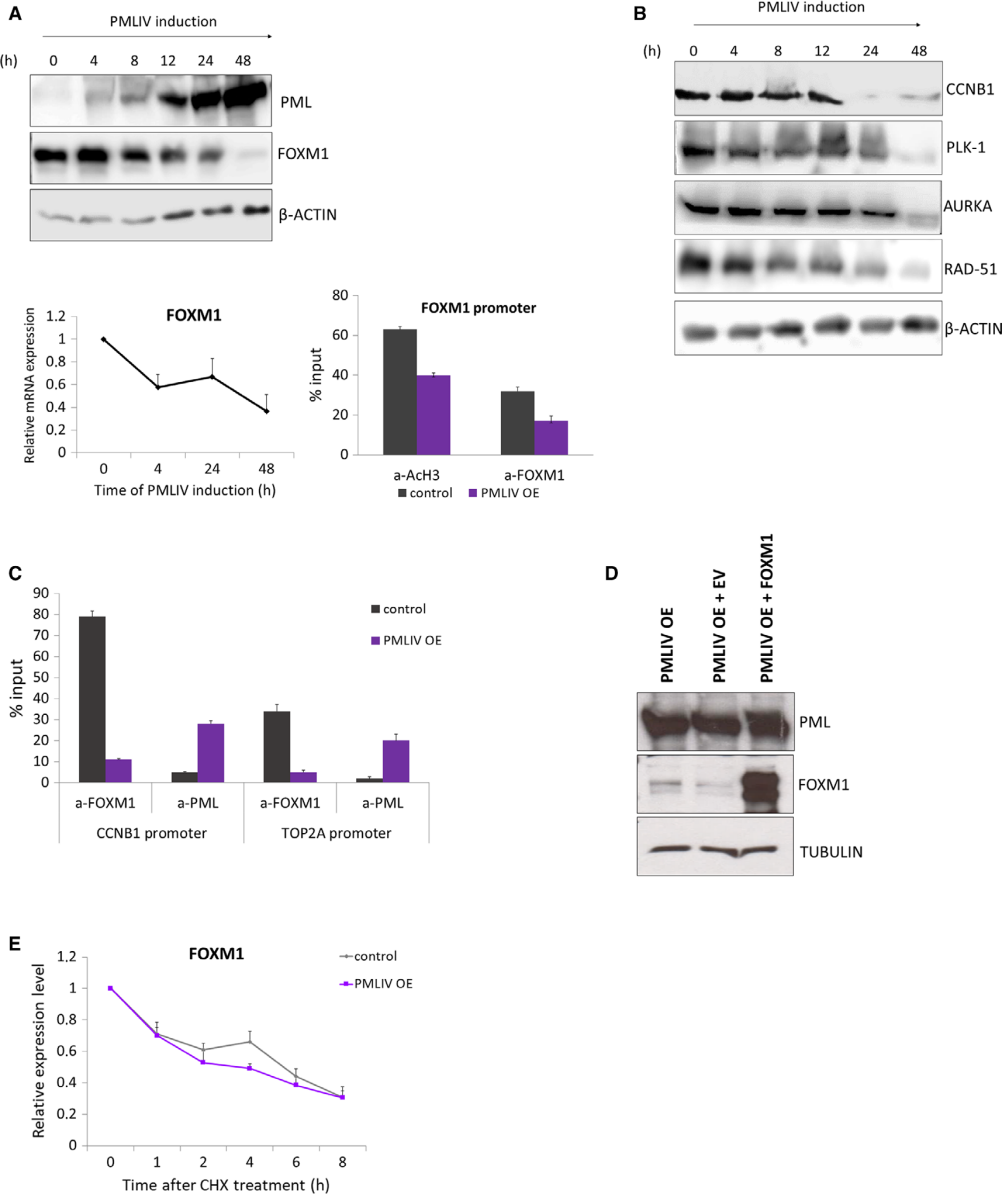
PMLIV OE downregulates FOXM1 expression. (A) Western blot analysis of MDA‐MB‐231 PMLIV OE cells for PML and FOXM1 during a time course of 48‐h PMLIV induction (upper panel). β‐actin was used as a loading control. FOXM1 relative mRNA levels upon PMLIV forced expression (lower panel). Error bars indicate mean + SD of *n* = 3 independent experiments. ChIP‐qPCR analysis for *FOXM1* promoter with antibodies against AcH3 and FOXM1 in control and PMLIV OE cells. Factor occupancy is expressed as % of input chromatin. Error bars represent mean + SD of *n* = 3 independent experiments. (B) Western blot analysis of MDA‐MB‐231 PMLIV OE cells for FOXM1 downstream targets during a time course of 48‐h PMLIV induction. (C) ChIP‐qPCR analysis for the *CCNB1* and *TOP2A* promoters with antibodies against FOXM1 and PML before and after PMLIV OE in MDA‐MB‐231 cells. Factor occupancy is expressed as % of input chromatin. Error bars indicate mean + SD of *n* = 3 independent experiments. (D) Western blot analysis for FOXM1 expression on lysates of MDA‐MB‐231 PMLIV OE cells transfected with FOXM1 under the control of the CMV promoter or empty vector. (E) MDA‐MB‐231 control and PMLIV OE cells were treated with 100 μmol·L^−1^ CHX, and FOXM1 protein levels were detected by western blotting. Densitometry was used to quantify the FOXM1 and β‐actin levels from which independent background readings were subtracted. Diagram depicting the relative expression levels of the ratios of FOXM1 to β‐actin relative to those at 0 h. Triplicate means and standard deviations are shown.

To further investigate the hypothesis that FOXM1 is a downstream signaling target of PML in breast cancer, we studied the effect of PMIV induction on FOXM1 protein and mRNA expression levels over a 48‐h time course. There was a significant decline in FOXM1 protein levels as early as 8 h after PMLIV ectopic expression, supporting that FOXM1 could be one of the primary cellular targets of PML. In agreement, FOXM1 mRNA was also reduced after 4 h of PMLIV overexpression (Fig. 4A). In addition, ChIP assays showed decreased AcH3 and FOXM1 recruitment on the *FOXM1* promoter upon PMLIV induction, corroborating with FOXM1 suppression at mRNA level. Consistent with the above, the reduction of FOXM1 expression was mirrored by the downregulation of FOXM1 targets, including cyclin B1, polo‐like kinase, Rad‐51, and AURKA (Fig. 4B). Using ChIP, we demonstrated that FOXM1 protein abundance directly correlated with its low recruitment to target promoters, such as *CCNB1* and *TOP2A*. Interestingly, PML was enriched on these promoters (Fig. 4C).

To investigate whether PML interferes with the FOXM1 auto‐regulatory loop, we transiently expressed a cytomegalovirus (CMV) promoter‐driven FOXM1 in MDA‐MB‐231 cells and we examined the effect of PMLIV on the endogenous and ectopic FOXM1 protein levels. Western blot analysis showed that endogenous FOXM1 was significantly downregulated upon PMLIV induction, whereas the protein level of ectopic FOXM1 did not change, supporting the notion that FOXM1 is primarily suppressed by PMLIV through a gene‐specific promoter transcriptional mechanism and not through protein or mRNA stability modulation (Fig. 4D). In addition, inhibition of protein synthesis by CHX treatment did not substantially shortened the half‐life of endogenous FOXM1 in PMLIV OE compared to the control cells, suggesting that the PMLIV‐mediated FOXM1 repression is predominantly independent of protein stability (Fig. 4E). We also studied whether FOXM1 is downregulated through protein degradation by the ubiquitin–proteasome pathway and tested the effect of the proteasome inhibitor MG132 treatment on the expression of endogenous FOXM1 before and after PMIV induction in MDA‐MB‐231 cells. Treatment with MG132 did not prevent the downregulation of FOXM1 protein levels by PMLIV OE, indicating that the downregulation of FOXM1 expression upon PMLIV induction is unlikely to be depended on proteasomal degradation (Fig. S3D).

### 3.5 PMLIV overexpression modulates FOXO3 transcriptional program

**Figure 5:**
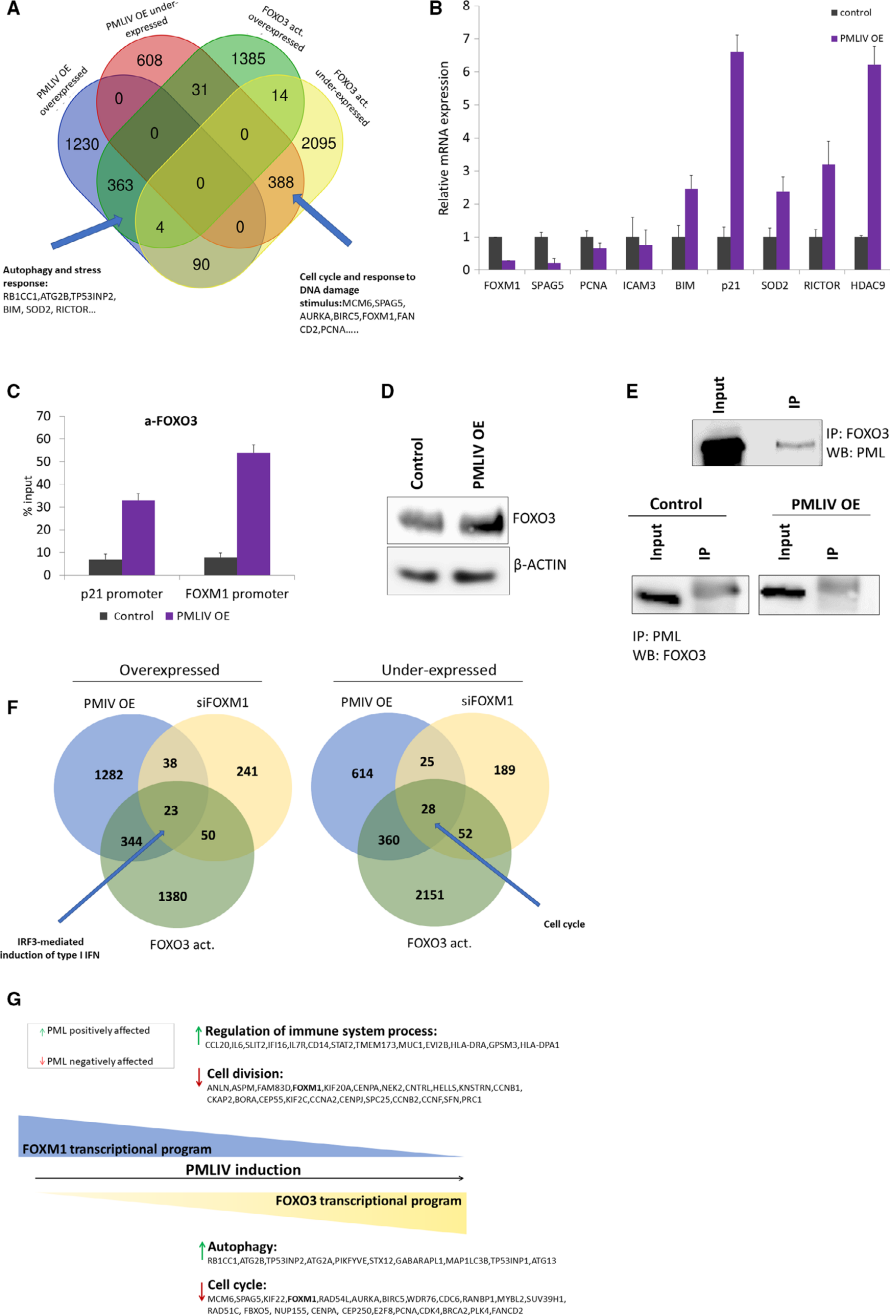
PMLIV OE modulates FOXO3 transcriptional program. (A) Venn diagram illustrating the overlap of PMLIV and FOXO3 targets in MDA‐MB‐231 cells and representative common genes. Datasets for constitutively active FOXO3 were retrieved from GEO (GSE113479). We noticed that 18 genes were common between FOXO3 overexpressed and under‐expressed genes. This is due to the different gene isoform probe sets on the microarray platform exhibiting different expression patterns between the same conditions. (B) Relative mRNA expression levels of known and PML common‐FOXO3 targets before and upon PMLIV induction. Error bars indicate + SD of three independent experiments (*n* = 3). (C) ChIP‐qPCR analysis for *p21* ^*Cip1*^ and *FOXM1* promoters with an antibody against FOXO3 in control and PMLIV OE MDA‐MB‐231 cells. Factor occupancy is expressed as % of input chromatin. Error bars indicate mean + SD of *n* = 3 independent experiments. (D) Western blot analysis of FOXO3 protein levels upon PMLIV forced expression. β‐actin was used as a loading control. (E) Co‐IP of PML and FOXO3 on lysates from transfected HEK293T cells OE PMLIV and FOXO3 (upper panel) and from lysates of MDA‐MB‐231 PMLIV OE cells (lower panel). (F) Venn diagrams depicting the common overexpressed and under‐expressed genes between PMLIV OE, siFOXM1, and constitutively active FOXO3 datasets. The common genes were submitted in g:profiler for functional enrichment analysis. (G) A schematic model illustrating the relationship between PML, FOXO3, and FOXM1 in breast cancer cells. PML acts as a regulator for opposing actions on the FOXO3 and FOXM1 activity balance. PMLIV induction results in activation of FOXO3 and inactivation of FOXM1 signaling. Acting on a subset of both common and unique target genes PML promotes divergent pathways that lead to cell growth arrest as well as to long‐term survival and stress resistance.

The above results identify FOXM1 as a novel PMLIV target. We next investigated whether PMLIV also affects FOXO3, another FKH transcription factor and an important negative regulator of FOXM1 (McGovern *et al*., 2009). Interestingly, FOXO3 was enriched in our transcriptomic data of PMLIV OE cells (Fig. 2D). Moreover, when we compared our list of DEGs by PMLIV induction with published microarray data of DEGs by constitutively active FOXO3 in MDA‐MB‐231 cells (GSE113479), we observed significant overlap between PMLIV and FOXO3 targets (Fig. 5A). Three hundred sixty‐seven common overexpressed genes were found between PMLIV OE and FOXO3 datasets, whereas 388 common were under‐expressed (among them FOXM1). The common under‐expressed genes of PMLIV OE and constitutively active FOXO3 were significantly enriched in cell cycle and response to DNA damage‐related GO terms, whereas the overexpressed in autophagy and stress response terms (Fig. S4A).

This analysis indicates that in addition to FOXM1, PMLIV DEGs overlap also with a large set of FOXO3 targets as revealed by the transcriptomic data and further validated by qPCR (Fig. 5B). FOXO3 targets for validation were selected based on the literature (Karadedou *et al*., 2012; Lam *et al*., 2013) and our overlap analysis. ChIP also confirmed FOXO3 potentiation, as FOXO3 recruitment to *FOXM1* and *p21* ^*Cip1*^ promoters was increased in PMLIV OE cells compared to the controls (Fig. 5C). Corroborating with the above results, immunofluorescent staining showed increased nuclear FOXO3 upon PMLIV induction (Fig. S4B).

To study the mechanism by which PMLIV potentiates FOXO3 activity, we first analyzed FOXO3 protein levels before and after PMLIV OE. Total FOXO3 protein levels did not significantly change, indicating that PML may post‐translationally modulate FOXO3 activity (Fig. 5D). We next asked whether FOXO3 also associates with PMLIV. Indeed, FOXO3 immunoprecipitated with PMLIV when both proteins were ectopically expressed in HEK293 cells (Fig. 5E). Moreover, endogenous FOXO3 interacted with PML in MDA‐MB‐231 cells before and after PMLIV induction (Fig. 5E). These results support that FOXO3 is involved in protein–protein interactions with PMLIV.

Immunofluorescence staining of endogenous FOXO3 or FOXM1 in parallel with PML in MDA‐MB‐231 cells showed that in agreement with the bulk western blot data, cells that expressed high levels of PML, even in the control state, completely or almost completely lack FOXM1, whereas cells with low PML expression had higher FOXM1 expression (Fig. S4C). In contrast, the same or slightly stronger FOXO3 expression corresponded to cells with high PML levels. Thus, PMLIV interferes with the FOXO3‐FOXM1 network not only through direct interaction with FOXM1 but also through potentiation of FOXO3 that in turn represses FOXM1 expression.

Given that FOXO3 antagonizes FOXM1‐dependent transcription (Lam *et al*., 2013) and that PMLIV OE deregulates FOXM1 and FOXO3 targets, we asked whether there is an overlap between PMLIV OE, siFOXM1, and constitutively active FOXO3 targets. Analysis of common genes of all three datasets showed that 23 overexpressed and 28 under‐expressed genes were enriched in IRF3‐mediated induction of type I IFN and cell cycle signatures, respectively (Fig. 5F). Taken into consideration the double (PMLIV OE‐siFOXM1 and PMLIV OE‐constitutively active FOXO3) and the triple overlaps (PMLIV OE‐siFOXM1‐FOXO3), it seems that PML regulates both common and distinct FOXO3‐FOXM1 gene subsets (Fig. 5G).

To further examine the role of PMLIV in MDA‐MB‐231 cells, we knocked down PML using lentiviruses constitutively expressing shRNA targeting all isoforms or specifically the PMLIV isoform only. Transduced cell pools were tested at early passage following puromycin selection. The efficiency of PML and PMLIV KD was assessed by immunofluorescence staining using an antibody that detects all PML isoforms. As expected, PML protein levels were strongly suppressed in total PML KD cells, whereas a slight reduction of the PML expression in the isoform‐specific PML KD, was observed, in agreement with our unpublished RNA‐seq data estimating that PMLIV represents about 25% of total PML RNA (Fig. S5A). To confirm that PMLIV was indeed specifically KD, we used PMLIV‐specific primers for qPCR analysis and showed similar degree of silencing in both total PML and PMLIV KD cells. *FOXM1* mRNA did not change significantly upon PML or PMLIV KD, whereas *FOXO3* mRNA level slightly decreased in both cases. Targets of FOXM1 and FOXO3 such as *TOP2A* and *PCNA* were upregulated, while the self‐renewal and EMT markers, *SOX2* and *ZEB1*, respectively, were downregulated in both total PML and PMLIV KD cells (Fig. S5B). Specific PMLIV KD elicited a more potent decrease of the growth rate and tumorsphere formation capacity relative to total PML KD in MDA‐MB‐231 cells (Fig. S5C). In addition, Annexin V‐FITC plus PI staining showed increased apoptosis in PMLIV KD and to a lesser extent in total PML KD compared to control cells (Fig. S5D). Neither total nor PMLIV KD cells showed changes of senescence‐associated β‐galactosidase staining (data not shown).

## 4 Discussion

The dual role of PML in cancer under cell‐specific contexts is intriguing (Mazza and Pelicci, 2013). The activity of PML is intrinsically linked to its interacting cofactors. Hence, understanding the discerning factors and networks that dictate PML's tumor‐suppressive or promoting role is essential not only for elucidating PML biology but also for providing insights into potential future therapeutics.

In this study, we addressed the role of PML in breast cancer cells. Our results propose that PMLIV overexpression inhibits the proliferation of breast cancer cells by concurrently regulating the oncogenic transcription factor FOXM1 and the tumor suppressor FOXO3 that acts upstream of FOXM1. Forced expression of PMLIV leads to decreased proliferation rate, clonogenicity, and sphere‐forming efficiency of MDA‐MB‐231 cells, as well as to deregulated cell cycle progression. Our data are in line with previous studies that link PML with cell cycle restriction through different mechanisms including regulation of the tumor suppressor pRB activity by promoting its PP1‐dependent dephosphorylation (Mu *et al*., 1997; Le *et al*., 1998; Wang *et al*., 1998; Regad *et al*., 2009), as well as p21 induction in p53‐dependent (Pearson *et al*., 2000) and p53‐independent manner (Cao *et al*., 2011).

Our transcriptomic and biochemical data connect for the first time PMLIV with the critical cell cycle regulator, FOXM1. We demonstrated that FOXM1 mRNA and protein levels as well as its downstream effectors negatively correlate with PMLIV induction. Accordingly, FOXM1 downregulation is consistent with the cell cycle arrest phenotype of MDA‐MB‐231 cells observed upon PMLIV induction. FOXM1 occupancy on its target promoters, for example, *CCNB1* and *TOP2A*, was reduced upon PMLIV induction reflecting its decreased transcriptional activity. Interestingly, PML was recruited to the above promoters implying that it may be directly involved in their suppression. In addition, the reported here physical interaction of PML with FOXM1′s DBD may further prevent FOXM1′s binding to its target promoters. Hence, PML can interfere with both FOXM1 gene expression and protein activity.

Promyelocytic leukemia may also interfere with other components of the FOXM1 network to indirectly regulate its expression. Our transcriptomic analysis shows that FOXO3, an upstream FOXM1 repressor, shares common target genes, including FOXM1, with PMLIV. Indeed, a subset of the FOXO3 transcriptional program is activated following PMLIV OE. This may involve a direct physical interaction, as we show here, in a way that resembles the deacetylation action of PML on the FOXO1 factor (Kitamura *et al*., 2005) but may also be indirect through modulation of various other PML targets that modify FOXO3 activity. Such a candidate is the AKT kinase that inactivates FOXO3 by phosphorylation and nuclear exclusion (Brunet *et al*., 1999). Interestingly, PML promotes inhibitory dephosphorylation of AKT at T308 and S473 by protein phosphatase 2 (PP2A) and PH domain and leucine‐rich repeat PP2A (PHLPP2) phosphatases, respectively (Trotman *et al*., 2006; Chatterjee *et al*., 2013). PMLIV OE cells have low S473 phosphorylation levels but no change of T308p hinting to AKT inhibition by PHLPP2 and thus increased FOXO3 activity (Fig. S4D).

To extend our results, we also examined the luminal type T47D cells, using the same approach. Although PMLIV induction inhibits the proliferation of both cell types, the cancer stem cell‐like activity, as measured by tumorsphere formation, is strongly inhibited in MDA‐MB‐231 but to a much lesser extent in T47D cells. Finally, preliminary transcriptomic data from PMLIV OE T47D cells (data not shown) support that in addition to a core of common, transcriptionally affected genes, many other genes show cell type specificity to ectopic PMLIV expression that may be related to context‐dependent effects of PML in tumor biology.

Other PMLIV OE‐enriched transcription factors, identified here, are E2F and NFY. The PML‐E2F connection has been previously described (Vernier *et al*., 2011). Unpublished data from our laboratory show that NFY‐A interacts with PMLIV and colocalizes in PML‐NBs thereby connecting PML with another cell cycle regulator. Overall, we suggest that the cell cycle arrest caused by PMLIV ectopic expression is mediated by the function of multiple and overlapping transcription factors that include the FOXO3‐FOXM1 axis.

To further explore the role of PMLIV in the above processes, we knocked down all PML or specifically isoform IV in MDA‐MB‐231 cells. RNA expression of *TOP2A, PCNA, SOX2, EZH2, ZEB1* genes associated with cell cycle, cancer cell ‘stemness’, and FOXM1‐FOXO activities was similarly affected in both cases. In spite of that, we found that specific reduction of the PMLIV variant had a stronger effect on cell proliferation, tumorsphere formation, and apoptosis than elimination of all isoforms that point to the possibility that other isoforms may counteract its antigrowth potential.

Other studies have shown that high PML levels correlate with poor prognosis in breast cancer patient sets, and PML KD compromises tumor growth and metastasis (Carracedo *et al*., 2012; Martin‐Martin *et al*., 2016; Ponente *et al*., 2017). Although total PML loss has been extensively studied both in cell and mouse knockout models, little is known about the roles of isoform‐specific loss with the exception of viral infection and immunity (Maroui *et al*., 2011; Ohsaki *et al*., 2016; Chen *et al*., 2018). Our results define PMLIV as the major prosurvival factor at least in this cancer context. A more precise determination of the relative PML isoform abundance in various clinical cancer datasets may help to better interpret the correlation of PML levels with patient survival that so far are based on total PML RNA levels. Further analysis is required to precisely define the PMLIV‐specific transcriptomic or proteomic targets in different cancer genetic backgrounds that mediate its cell survival protective/suppressive role and may be counteracted by other PML isoforms.

## 5 Conclusions

By the concurrent activation of FOXO3 and inactivation of FOXM1 signaling, PMLIV promotes divergent pathways that lead to cell growth arrest and at the same time favor long‐term survival and stress resistance. These contrasting activities may be essential in different cancer contexts that carry or evolve to different genetic and epigenetic background and may provide a mechanistic basis for the seemingly dual role of PML as tumor promoting or suppressing gene. Hence, we provide evidence of a novel and potentially targetable PML‐FOXO3‐FOXM1 axis that may be of benefit in particular settings. Our findings expand our understanding on the transcriptional networks that govern breast cancer and may have important implications for therapeutic interventions.

## Conflict of interest

The authors declare no conflicts of interest.

## Author contributions

NS designed and performed experiments, data analysis, discussion, and writing; PA and TM contributed to the experimental work and discussion; AK and CN performed microarrays and bioinformatic analysis; EL and AK contributed to discussion and writing; JP supervised the study and contributed to writing.

## Supporting information


**Fig. S1.** PMLIV represses the proliferation of breast cancer cells.
**Fig. S2.** Genome wide analysis of control and PMLIV OE MDA‐MB‐231 cells.
**Fig. S3.** FOXM1 specifically interacts with PMLIV and colocalizes in the PMLIV‐NBs.
**Fig. S4.** PMLIV modulates FOXO3 transcriptional program.
**Fig. S5.** Effect of PML KD and specific PMLIV KD in MDA‐MB‐231 cells.

**Table S1.** Primer sets used for qPCR.

**Table S2.** DEGs upon PMLIV induction.

## Data Availability

Microarray datasets have been deposited in GEO under accession number GSE119583.
