# Supplementary material for: Promyelocytic leukemia protein (PML) controls breast cancer cell proliferation by modulating Forkhead transcription factors
Source: Mol Oncol. 2019 May 16;13(6):1369–87. doi: 10.1002/1878-0261.12486 (PMC6547613; doi:10.1002/1878-0261.12486)
Supplement: Supplementary file 1 — Fig. S1. PMLIV represses the proliferation of breast cancer cells. Fig. S2. Genome wide analysis of control and PMLIV OE MDA‐MB‐231 cells. Fig. S3. FOXM1 specifically interacts with PMLIV and colocalizes in the PMLIV‐NBs. Fig. S4. PMLIV modulates FOXO3 transcriptional program. Fig. S5. Effect of PML KD and specific PMLIV KD in MDA‐MB‐231 cells. [file MOL2-13-1369-s001.pdf]

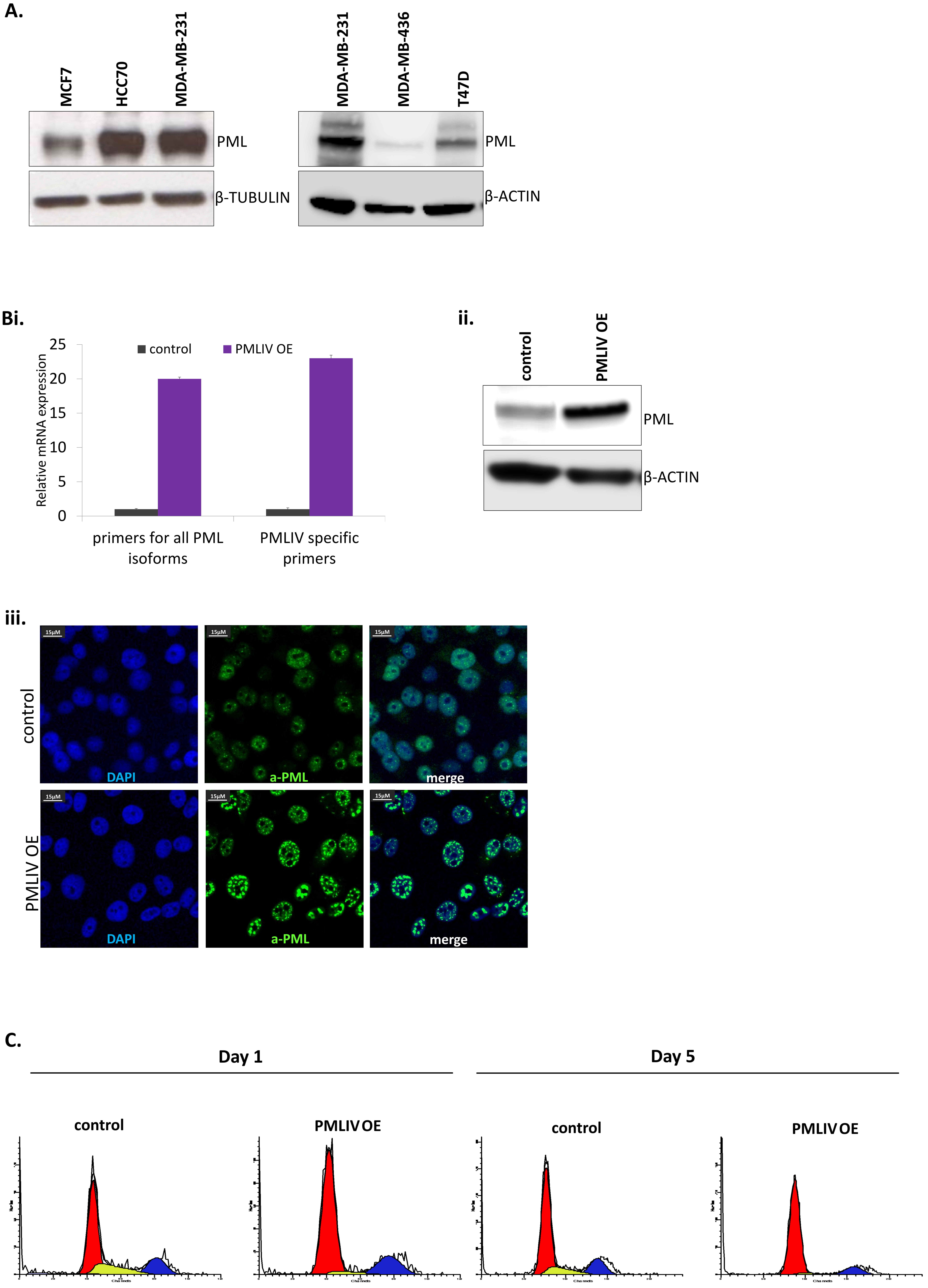
A.
MCF7
HCC70
MDA-MB-231
PML
β-TUBULIN
MDA-MB-231
MDA-MB-436
T47D
PML
β-ACTIN
Bi.
control
PMLIV OE
Relative mRNA expression
25
20
15
10
5
0
primers for all PML isoforms
PMLIV specific primers
ii.
control
PMLIV OE
PML
β-ACTIN
iii.
control
PMLIV OE
15μM
DAPI
a-PML
merge
C.
Day 1
Day 5
control
PMLIV OE
control
PMLIV OE

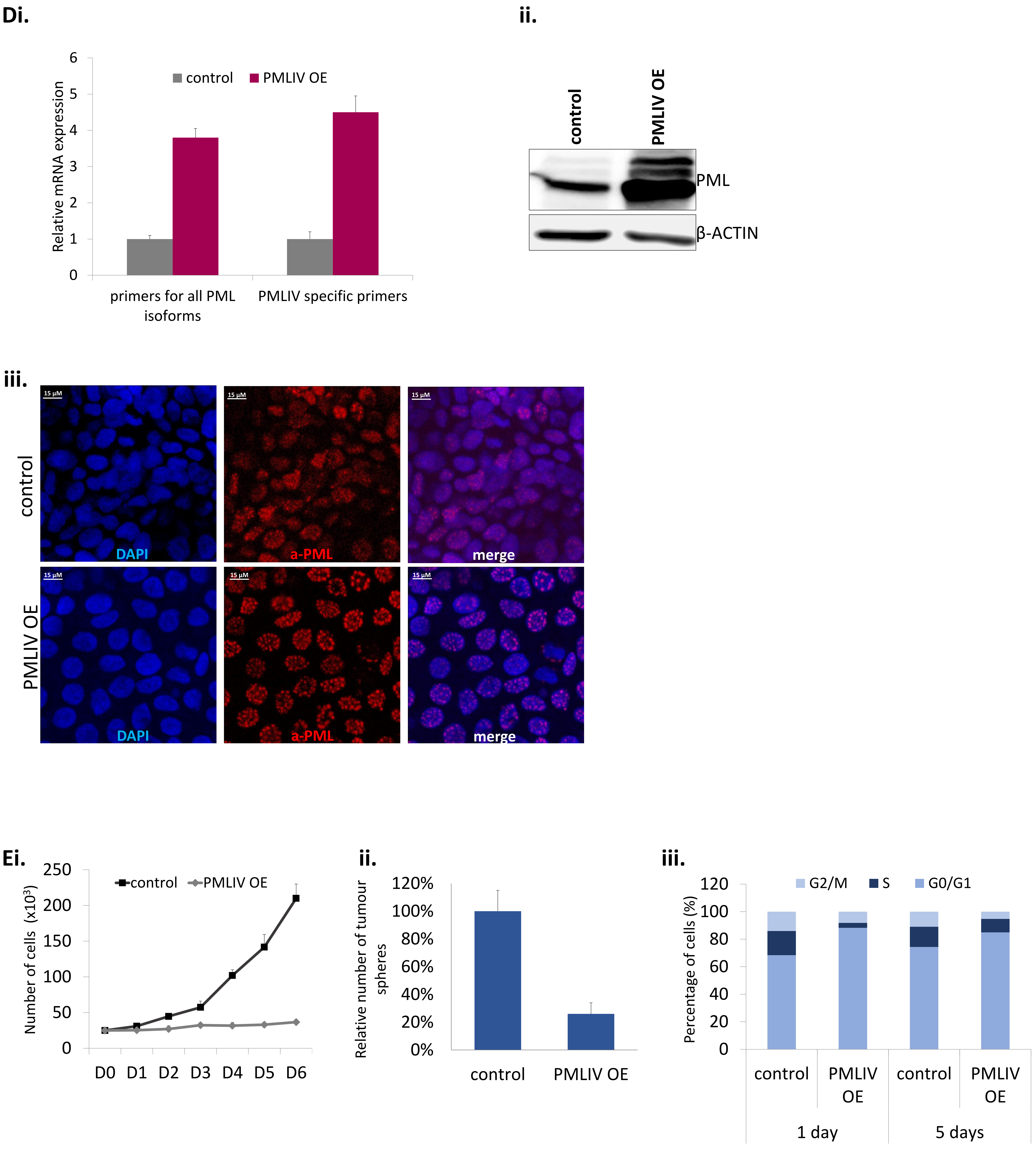

Figure S1 (related to Figure 1). PMLIV represses the proliferation of breast cancer cells. A. PML protein levels in a panel of breast cancer cell lines. β-actin and β-tubulin were used as loading controls. B. Assessment of PMLIV overexpression before and after doxycycline treatment in MDA-MB-231 cells. i. Relative mRNA expression levels of PML in control and PMLIV overexpressing (OE) cells. The mRNA level was checked with primers that detect all PML isoforms and with PMLIV specific primers. Error bars indicate +SD in three independent experiments (n=3). ii. PML protein levels of control and PMLIV OE cells. β-actin was used as a loading control. iii. Immunofluorescence staining for PML in control and PMLIV OE MDA-MB-231 cells (Scale bar, 15μM). C. Cell cycle phase distribution of control and PMLIV OE cells after 1 and 5 days of PMLIV induction. D. Assessment of PMLIV overexpression before and after doxycycline treatment in T47D cells. i. Relative mRNA expression levels of PML in control and PMLIV overexpressing (OE) cells. The mRNA level was checked with primers that detect all PML isoforms and with PMLIV specific primers. Error bars indicate +SD in three independent experiments (n=3). ii. PML protein levels of control and PMLIV OE cells. β-actin was used as a loading control. iii. Immunofluorescence staining for PML in control and PMLIV OE T47D cells (Scale bar, 15μM). E. i. Cell growth of control and PMLIV overexpressing (PMLIV OE) T47D cells. Data represent the mean +SD of three independent experiments (n=3). ii. Tumorsphere formation of control and PMLIV OE MDA-MB-231 cells. Results are shown as mean + SD of three independent experiments (n=3). iii. Cell cycle analysis of control and PMLIV OE cells stained with propidium iodide (PI) and analyzed using flow cytometry after 1 and 5 days of PMLIV induction.

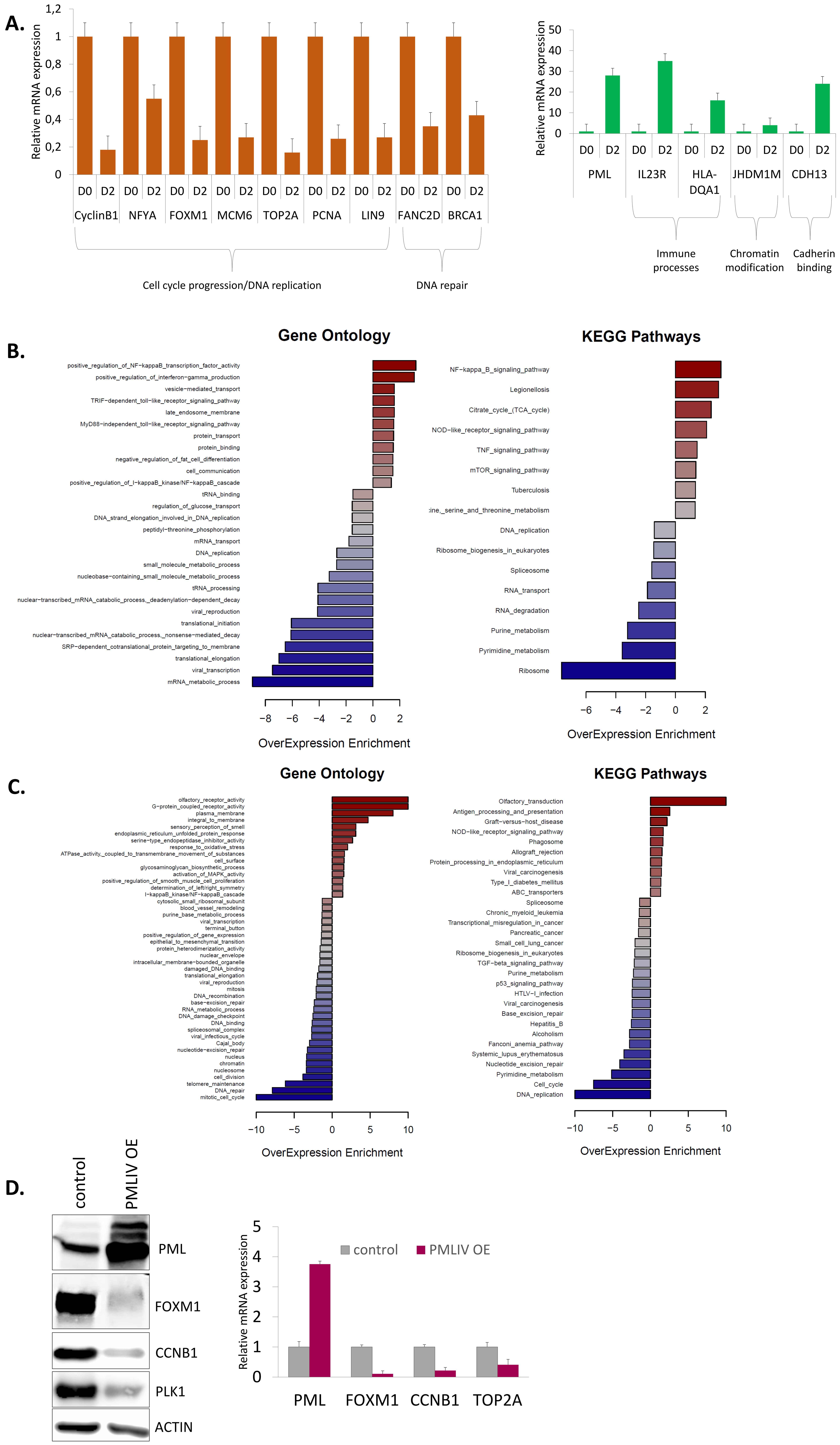
A.
Relative mRNA expression
1,2
1
0,8
0,6
0,4
0,2
0
D0 D2 D0 D2 D0 D2 D0 D2 D0 D2 D0 D2 D0 D2 D0 D2 D0 D2
CyclinB1 NFYA FOXM1 MCM6 TOP2A PCNA LIN9 FANC2D BRCA1
Cell cycle progression/DNA replication
DNA repair
Relative mRNA expression
50
40
30
20
10
0
D0 D2 D0 D2 D0 D2 D0 D2 D0 D2
PML IL23R HLA-DQA1 JHDM1M CDH13
Immune processes
Chromatin modification
Cadherin binding
B.
Gene Ontology
positive_regulation_of_NF−kappaB_transcription_factor_activity
positive_regulation_of_interferon−gamma_production
vesicle−mediated_transport
TRIF−dependent_toll−like_receptor_signaling_pathway
late_endosome_membrane
MyD88−independent_toll−like_receptor_signaling_pathway
protein_transport
protein_binding
negative_regulation_of_fat_cell_differentiation
cell_communication
positive_regulation_of_I−kappaB_kinase/NF−kappaB_cascade
tRNA_binding
regulation_of_glucose_transport
DNA_strand_elongation_involved_in_DNA_replication
peptidyl−threonine_phosphorylation
mRNA_transport
DNA_replication
small_molecule_metabolic_process
nucleobase−containing_small_molecule_metabolic_process
tRNA_processing
nuclear−transcribed_mRNA_catabolic_process,_deadenylation−dependent_decay
viral_reproduction
translational_initiation
nuclear−transcribed_mRNA_catabolic_process,_nonsense−mediated_decay
SRP−dependent_cotranslational_protein_targeting_to_membrane
translational_elongation
viral_transcription
mRNA_metabolic_process
−8 −6 −4 −2 0 2
OverExpression Enrichment
KEGG Pathways
NF−kappa_B_signaling_pathway
Legionellosis
Citrate_cycle_(TCA_cycle)
NOD−like_receptor_signaling_pathway
TNF_signaling_pathway
mTOR_signaling_pathway
Tuberculosis
ine,_serine_and_threonine_metabolism
DNA_replication
Ribosome_biogenesis_in_eukaryotes
Spliceosome
RNA_transport
RNA_degradation
Purine_metabolism
Pyrimidine_metabolism
Ribosome
−6 −4 −2 0 2
OverExpression Enrichment
C.
Gene Ontology
olfactory_receptor_activity
G−protein_coupled_receptor_activity
plasma_membrane
integral_to_membrane
sensory_perception_of_smell
endoplasmic_reticulum_unfolded_protein_response
serine−type_endopeptidase_inhibitor_activity
response_to_oxidative_stress
ATPase_activity,_coupled_to_transmembrane_movement_of_substances
cell_surface
glycosaminoglycan_biosynthetic_process
activation_of_MAPK_activity
positive_regulation_of_smooth_muscle_cell_proliferation
determination_of_left/right_symmetry
I−kappaB_kinase/NF−kappaB_cascade
cytosolic_small_ribosomal_subunit
blood_vessel_remodeling
purine_base_metabolic_process
viral_transcription
terminal_button
positive_regulation_of_gene_expression
epithelial_to_mesenchymal_transition
protein_heterodimerization_activity
nuclear_envelope
intracellular_membrane−bounded_organelle
damaged_DNA_binding
translational_elongation
viral_reproduction
mitosis
DNA_recombination
base−excision_repair
RNA_metabolic_process
DNA_damage_checkpoint
DNA_binding
spliceosomal_complex
viral_infectious_cycle
Cajal_body
nucleotide−excision_repair
nucleus
chromatin
nucleosome
cell_division
telomere_maintenance
DNA_repair
mitotic_cell_cycle
−10 −5 0 5 10
OverExpression Enrichment
KEGG Pathways
Olfactory_transduction
Antigen_processing_and_presentation
Graft−versus−host_disease
NOD−like_receptor_signaling_pathway
Phagosome
Allograft_rejection
Protein_processing_in_endoplasmic_reticulum
Viral_carcinogenesis
Type_I_diabetes_mellitus
ABC_transporters
Spliceosome
Chronic_myeloid_leukemia
Transcriptional_misregulation_in_cancer
Pancreatic_cancer
Small_cell_lung_cancer
Ribosome_biogenesis_in_eukaryotes
TGF−beta_signaling_pathway
Purine_metabolism
p53_signaling_pathway
HTLV−I_infection
Viral_carcinogenesis
Base_excision_repair
Hepatitis_B
Alcoholism
Fanconi_anemia_pathway
Systemic_lupus_erythematosus
Nucleotide_excision_repair
Pyrimidine_metabolism
Cell_cycle
DNA_replication
−10 −5 0 5 10
OverExpression Enrichment
D.
control
PMLIV OE
PML
FOXM1
CCNB1
PLK1
ACTIN
Relative mRNA expression
5
4
3
2
1
0
control
PMLIV OE
PML FOXM1 CCNB1 TOP2A

E.

Comparison	Target genes PMLIVOE/siFOXM1	Common genes (intersect)	Unique genes (union)	Jaccard index (intersect)	Mean Jaccard indexes (10.000 permutations)	Z-scores	p-value
Overexpressed genes	1687/352	61	1979	0.03	0.013	7.3	1.25E-13
Under-expressed genes	1027/294	53	1269	0.04	0.01	11.2	0

Under-expressed genes

GO.ID	Description	p.Value
GO:0051301	cell division	1.87E-15
GO:0022402	cell cycle process	7.45E-14
GO:1903047	mitotic cell cycle process	1.48E-12
GO:0007049	cell cycle	1.89E-11
GO:0000278	mitotic cell cycle	5.45E-11
GO:0000226	microtubule cytoskeleton organization	4.20E-08
GO:0007017	microtubule-based process	4.23E-06
GO:0044770	cell cycle phase transition	6.01E-06
GO:0051726	regulation of cell cycle	9.88E-06
GO:0098813	nuclear chromosome segregation	1.44E-05

Overexpressed genes

GO.ID	Description	p.Value
GO:0002682	regulation of immune system process	8.50E-04
GO:0051240	positive regulation of multicellular organismal process	1.21E-03
GO:0002684	positive regulation of immune system process	1.24E-03
GO:0051239	regulation of multicellular organismal process	3.29E-03
GO:0034097	response to cytokine	3.44E-03
GO:0048583	regulation of response to stimulus	3.61E-03
GO:0071345	cellular response to cytokine stimulus	6.91E-03
GO:0019221	cytokine-mediated signaling pathway	9.06E-03
GO:0001819	positive regulation of cytokine production	0.00924
GO:0001816	cytokine production	0.0151

Figure S2 (related to Figure 2). Genome wide analysis of control and PMLIV OE MDA-MB-231 cells. A. Relative mRNA levels of selected differentially expressed genes upon PMLIV OE. Error bars indicate +SD of three independent experiments (n=3). B. Bar charts depicting GO categories (left panel) and KEGG pathways (right panel) that are significantly enriched in overexpressed and under-expressed genes upon PMLIV forced expression in monolayer MDA-MB-231 cells (RNEA analysis). C. Bar charts depicting GO categories (left panel) and KEGG pathways (right panel) that are significantly enriched in overexpressed and under-expressed genes upon PMLIV forced expression in MDA-MB-231 mammospheres (RNEA analysis). D. Western blot analysis and relative mRNA expression levels of cell cycle regulators in T47D control and PMLIV OE cells. Error bars represent +SD of three independent experiments (n=3). E. Table showing the Jaccard similarity indexes calculated to assess the overlap for both over/under-expressed genes (upper table). The top 10 GO categories (g:Profiler) of under-expressed and overexpressed genes commonly affected by PMLIV OE and FOXM1 KD.

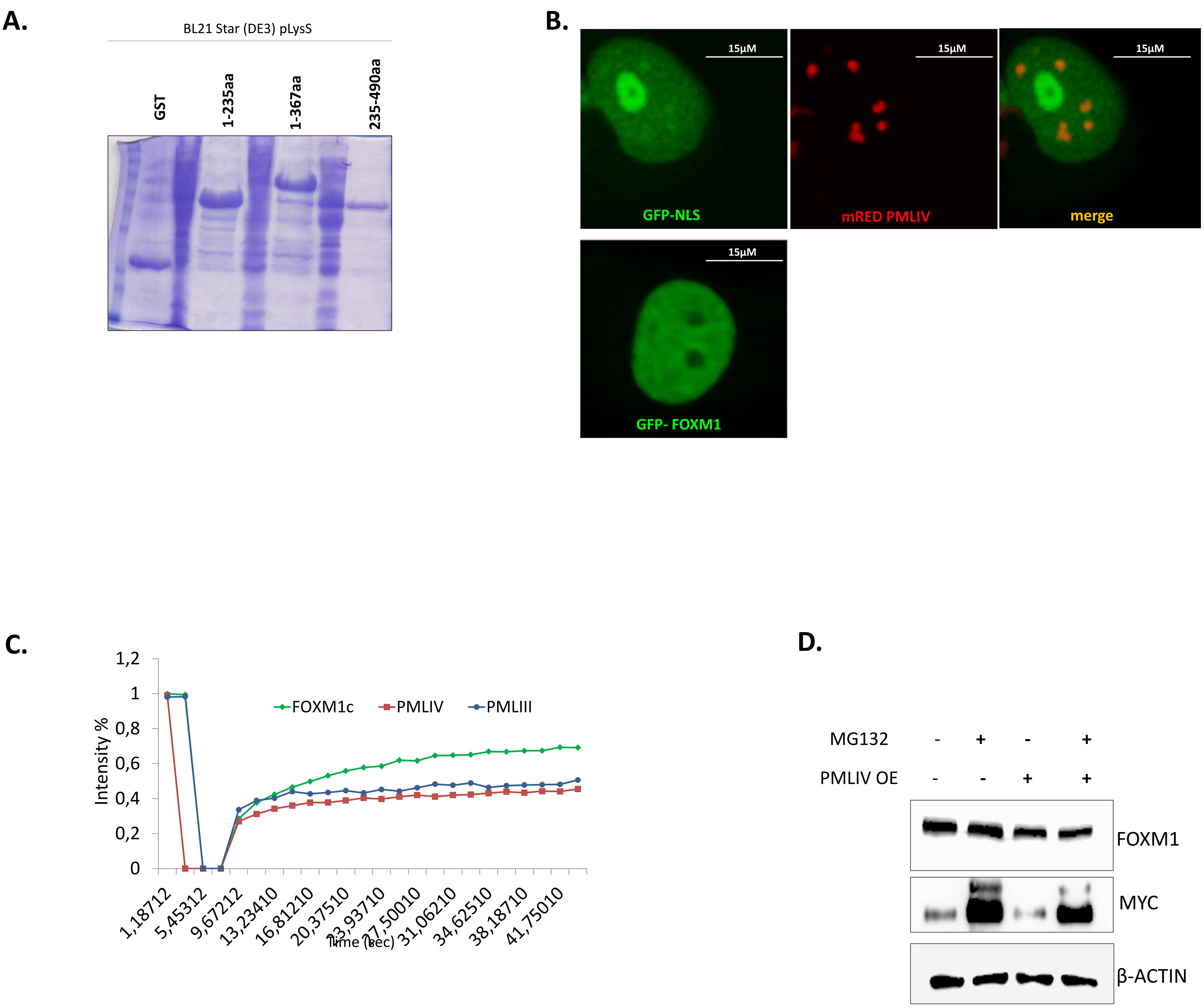

Figure S3 (related to Figure 3 and 4). FOXM1 specifically interacts with PMLIV and co-localizes in the PMLIV-NBs. A. Coomassie staining of GST-FOXM1 fusion proteins after their heterologous expression in BL21 Star (DE3) pLysS. B. Subnuclear localization of GFP-FOXM1 and GFP-NLS in Cos-7 cells served as controls (Scale bar, 15µM). C. FRAP experiment with transiently expressed GFP-FOXM1, mRED-PMLIV or mRED-PMLIII. D. Western blot analysis for FOXM1 and MYC in control and MDA-MB-231 PMIV OE treated with the proteasome inhibitor 10µM MG132 (InvivoGen) for 6 h. Myc was used as a positive control for MG132 treatment.

A.

Comparison	Target genes PMLIVOE/siFOXM1	Common genes (intersect)	Unique genes (union)	Jaccard index (intersect)	Mean Jaccard indexes (10.000 permutations)	Z-scores	p-value
Overexpressed genes	1687/1797	367	3118	0.11	0.03	26.2	0
Under-expressed genes	1027/2591	388	3231	0.12	0.03	31.5	0

Under-expressed genes

GO.ID	Description	p.Val
GO:0007049	cell cycle	1.55E-63
GO:0022402	cell cycle process	3.11E-60
GO:1903047	mitotic cell cycle process	7.22E-52
GO:0000278	mitotic cell cycle	8.36E-48
GO:0006260	DNA replication	6.26E-46
GO:0006259	DNA metabolic process	9.01E-46
GO:0006261	DNA-dependent DNA replication	3.45E-40
GO:0051276	chromosome organization	7.41E-37
GO:0044770	cell cycle phase transition	6.44E-34
GO:0006974	cellular response to DNA damage stimulus	1.29E-29

Overexpressed genes

GO.ID	Description	p.Value
GO:0000045	autophagosome assembly	2.55E-05
GO:1905037	autophagosome organization	3.85E-05
GO:0010033	response to organic substance	1.36E-04
GO:0035556	intracellular signal transduction	3.39E-04
GO:0071310	cellular response to organic substance	8.44E-04
GO:0016236	macroautophagy	9.86E-04
GO:0070647	protein modification by small protein conjugation or removal	1.40E-03
GO:0009968	negative regulation of signal transduction	2.36E-03
GO:0036211	protein modification process	2.41E-03
GO:0006464	cellular protein modification process	2.41E-03

B.

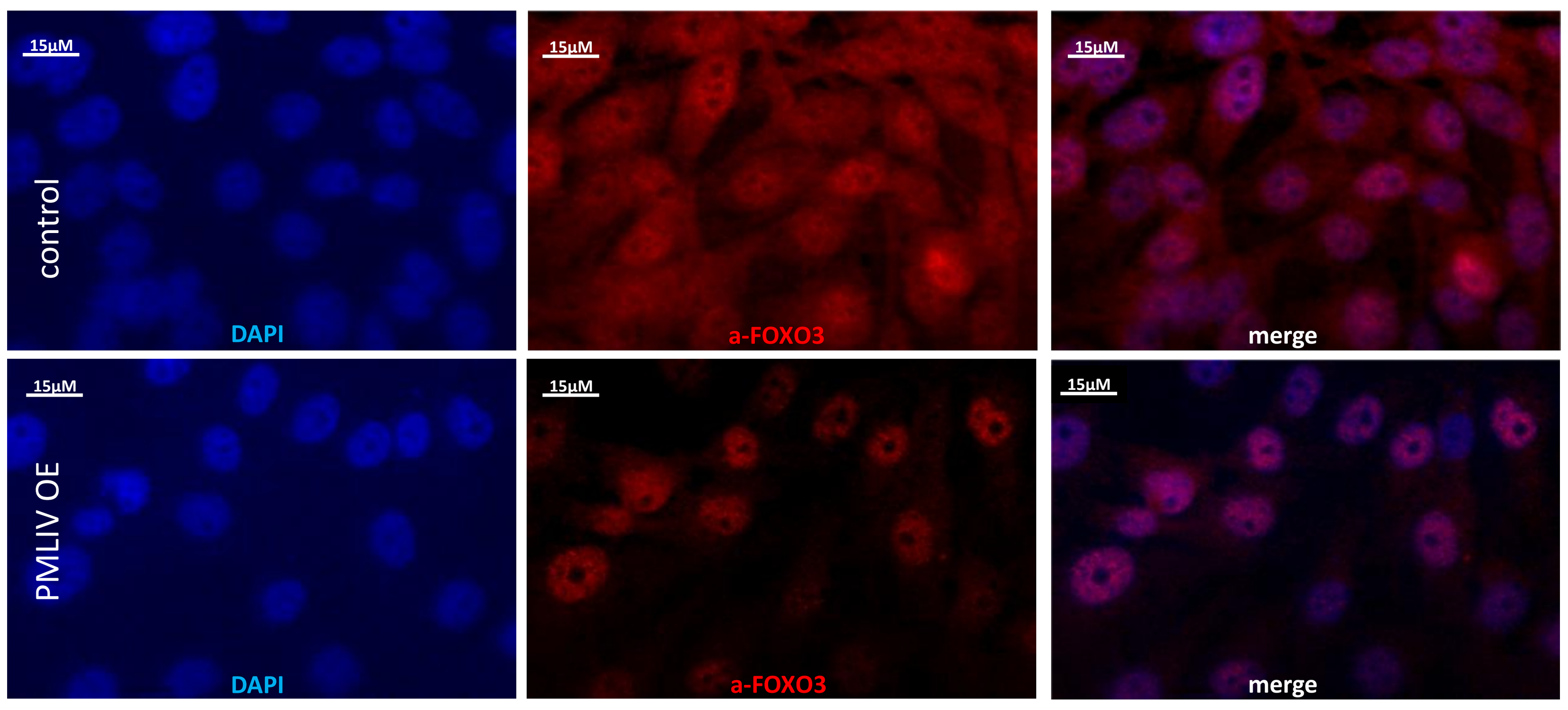

C.

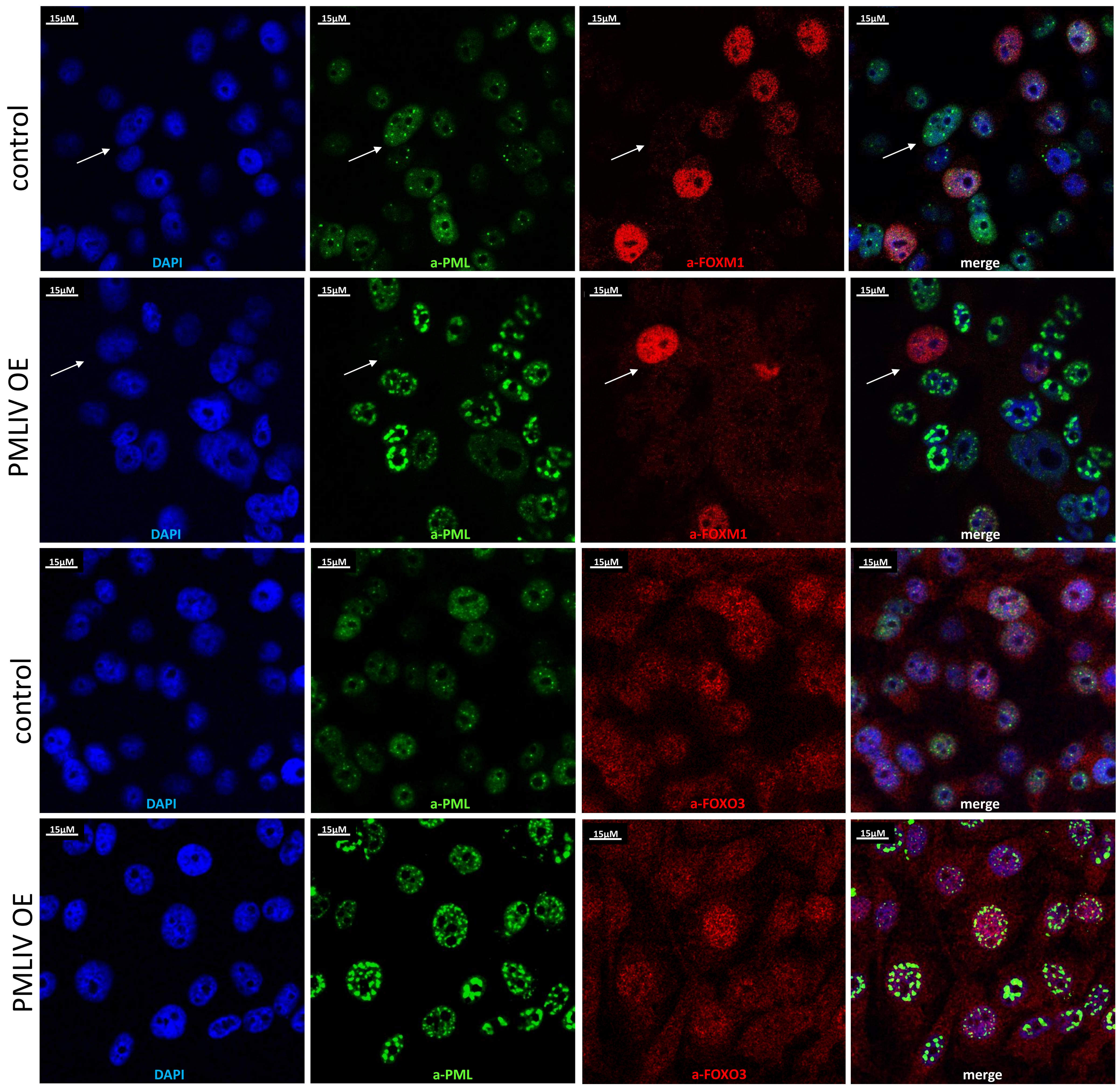

D.

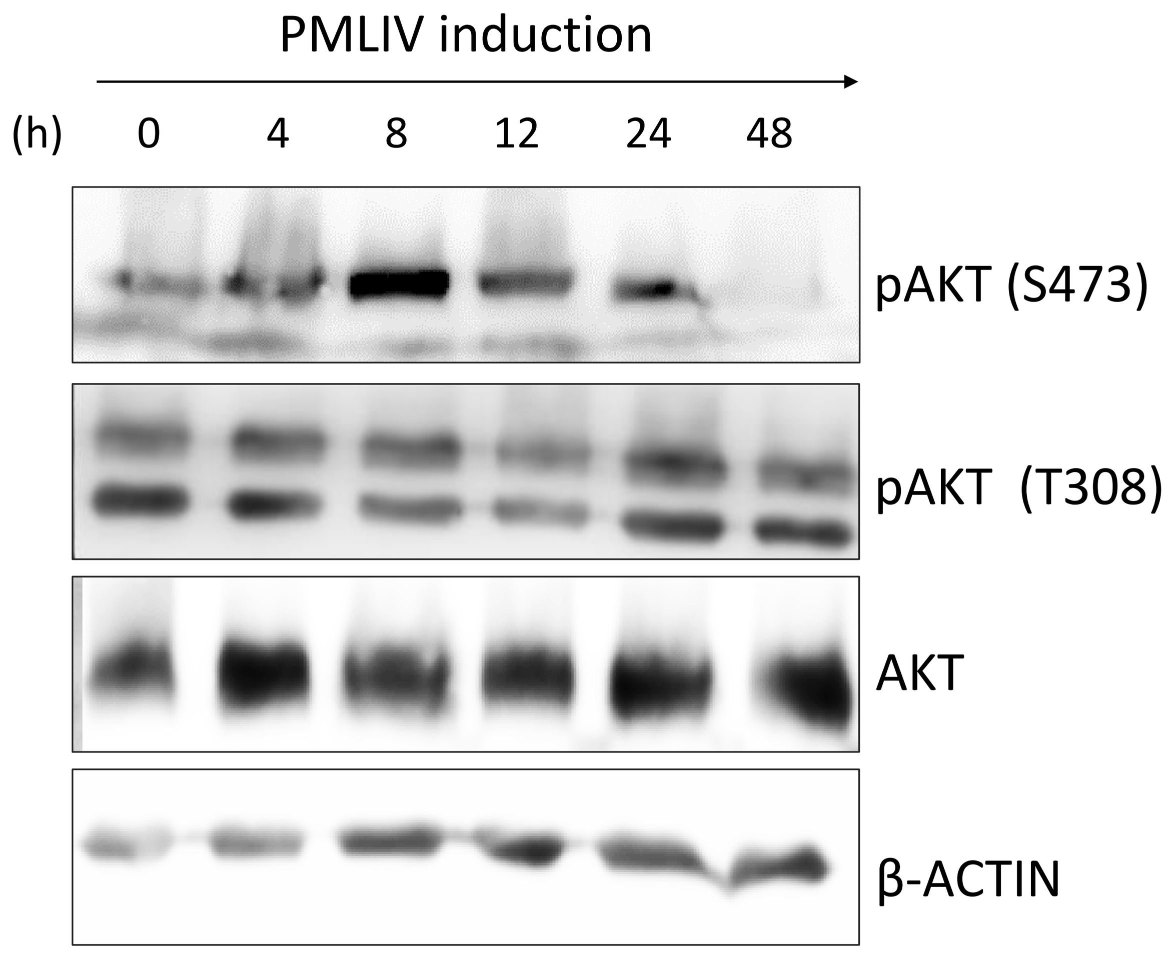

Figure S4 (related to Figure 5). PMLIV modulates FOXO3 transcriptional program. A. Table showing the calculated Jaccard similarity indexes computed to assess the degree of overlap for both over/under-expressed genes (upper panel). The top 10 GOs (g:Profiler) for under-expressed and overexpressed genes commonly affected by PMLIV OE and FOXO3 constitutive activation. B. Immunofluorescence staining for FOXO3 before and after PMLIV induction (Scale bar, 15μM). C. Immunofluorescence staining for PML and FOXM1 or FOXO3 in control and PMLIV OE MDA –MB-231 cells. Arrows indicate nuclei with strong or weak PML expression and the corresponding FOXM1 or FOXO3 levels (Scale bar, 15μm). D. Western blot analysis of MDA- MB-231 PMLIV OE cells for pAKT (S473, T308) and AKT over a 48 h time course PMLIV induction. β-actin was used as a loading control.

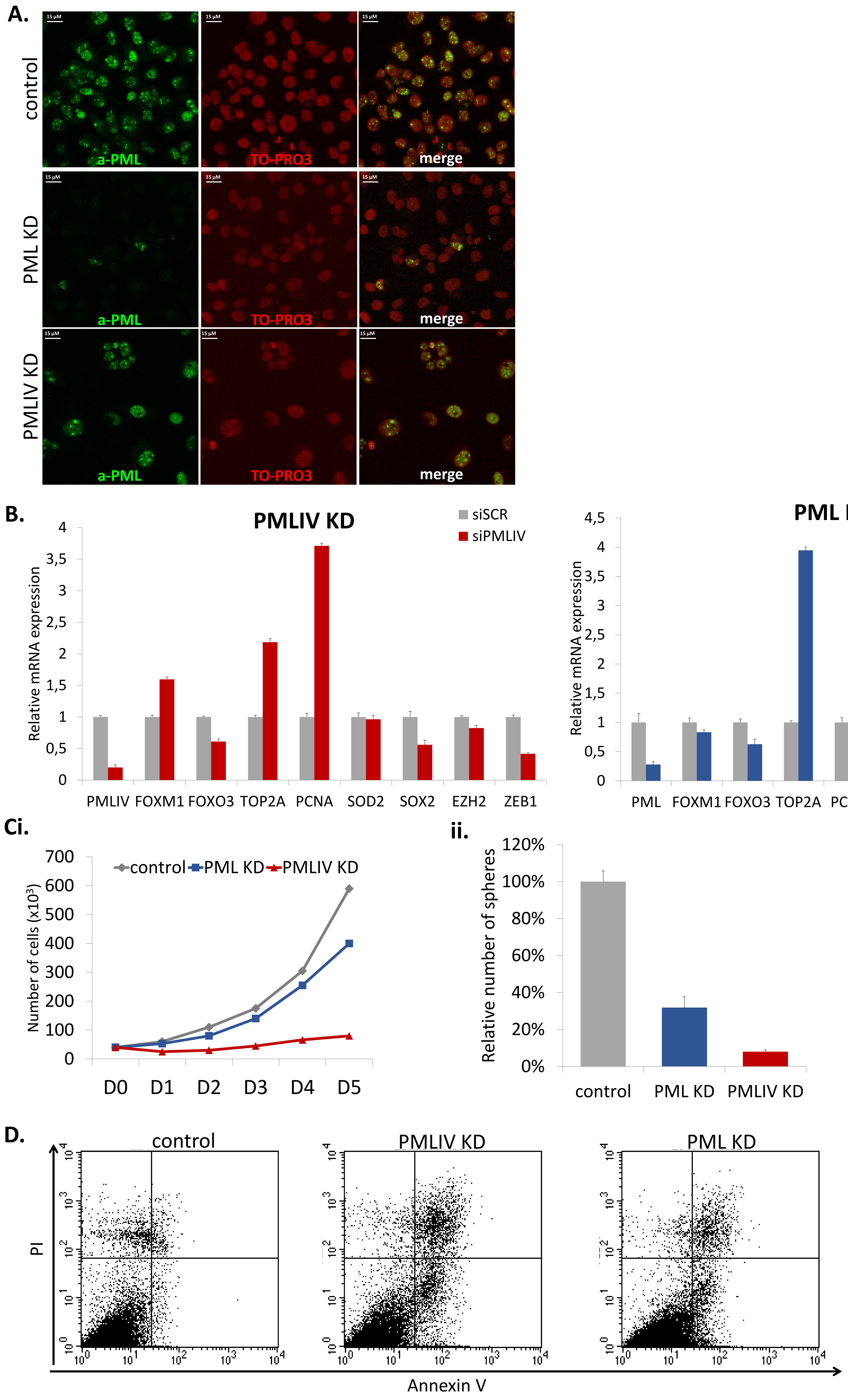

Figure S5: Effect of PML KD and specific PMLIV KD in MDA-MB-231 cells. A. Immunofluorescence staining for PML in control and PML or PMLIV KD MDA-MB-231 cells (Scale bar, 15μM). B. Relative mRNA expression of FOXM1, FOXO3, DNA replication and cancer stem cell markers upon control and PML KD or PMLIV KD in MDA-MB-231 cells. Error bars indicate +SD in three independent experiments (n=3). Ci. Cell growth of MDA-MB-231 cells upon PML and PMLIV silencing. ii. Tumorsphere formation in PML KD or PMLIV KD MDA-MB-231 cells. Results are shown as mean + SD of three independent experiments (n=3). D. Apoptotic cells after total PML KD or specific PMLIV KD were analyzed using flow cytometry after Annexin V-PI labeling. The lower right (LR) quadrant corresponds to the early to early-mid apoptotic cells, whereas the upper right (UR) quadrant represents cells at mid to mid-late apoptotic phases.
