# Supplementary material for: Promyelocytic leukemia protein (PML) controls breast cancer cell proliferation by modulating Forkhead transcription factors
Source: Mol Oncol. 2019 May 16;13(6):1369–87. doi: 10.1002/1878-0261.12486 (PMC6547613; doi:10.1002/1878-0261.12486)
Supplement: Supplementary file 2 — Table S1. Primer sets used for qPCR. [file MOL2-13-1369-s002.docx]

**Table S1: Primer sets used for qPCR.**

| PML | F: 5' CCCTGGATAACGTCTTTTTCG 3' |
| --- | --- |
|  | R: 5' GGAGCTGCTCGCACTCAAAGC 3' |
| PMLIV | F: 5’ CCAGGAGAACCCACTTTCA 3’ |
|  | R: 5’ AGCTCGGAAGACTCAGATG 3’ |
| ACTIN B | F: 5' CCTGTACGCCAACACAGTG 3' |
|  | R: 5' ATACTCCTGCTTGCTGATCC 3' |
| FOXM1 | F: 5' ATTGGACCAGGTGTTTAAGCC 3' |
|  | R: 5'GGTAGCAGTGGCTTCATCTT 3' |
| FOXO3 | F: 5’ TCTACGAGTGGATGGTGCGTT 3’ |
|  | R: 5’ CGACTATGCAGTGACAGGTTGTG 3’ |
| CCNB1 | F: 5′-GCAAGCAGTCAGACCAAAAT-3′ |
|  | R: 5′-CATGAACCGATCAATAATGG-3′ |
| TOP2A | F: 5'-TCAAACGGAATGACAAGCGA-3' |
|  | R: 5'-ATGGGCTGCAAGAGGTTTAG-3' |
| NFYA | F: 5'-TCAATTCAGGAGGGATGGTC-3' |
|  | R: 5'-ACGGTGGTATTGTTTGGCAT-3' |
| PCNA | F: 5'-TTTCCTGTGCAAAAGACGGA-3' |
|  | R: 5'-CCGTTGAAGAGAGTGGAGTGG-3' |
| MCM6 | F: 5'-TGGTGACCCAAGTACAGCTA-3' |
|  | R: 5'-CCGCACGTCCATCTTATCAA-3' |
| BRCA1 | F: 5'-TGTCTCCACAAAGTGTGACCA-3' |
|  | R: 5'-GCACGGTTTCTGTAGCCCAT-3' |
| FANCD2 | F: 5'-GACTCACCCAACATGTGCCT-3' |
|  | R: 5'-AAATGCAACCATCAGTGCCAG-3' |
| CCND1 | F: 5'-CCTCGGTGTCCTACTTCAAA-3' |
|  | R: 5'-TGTAGATGCACAGCTTCTCG-3' |
| CDH13 | F: 5'-AACGACAAGCTACGCTATGA-3' |
|  | R: 5'-TTCTGCCATATCTTCCGCAT-3' |
| p21 | F: 5'-CCGCTCTACATCTTCTGCCTTAGTC-3' |
|  | R: 5'-AACCTCTCATTCAACCGCCTAGTT-3' |
| LIN-9 | F: 5'-ATTCGAACCTCCCTCGCAA-3' |
|  | R: 5'-GAAAGGCATTTCCACAGCAG-3' |
| BIM | F: 5'-ATCATCGCGGTATTCGGTTC-3' |
|  | R: 5'- TCAGAAGGTTGCTTTGCCAT-3' |
| RICTOR | F: 5'-TGGATCTGACCCGAGAACCT-3' |
|  | R: 5'-CTCGCACTTCTTTTGCTTCA-3' |
| SOD2 | F: 5'-CTGGACAAACCTCAGCCCTA-3' |
|  | R: 5'-CTGATTTGGACAAGCAGCAA-3' |
| HDAC9 | F: 5’-GGTGGACAGTGACACCATTT-3’ |
|  | R: 5’-TGGATTCTTCAGCGTGATGG-3’ |
| IL23R | F: 5'-GAAAGCTGCCTTGCAATCTG-3' |
|  | R: 5'-TCCACCTTCGGGACCTTAAT-3' |
| HLA-DQA | F: 5'-TCTGATGTGTCCCTCACAGC-3' |
|  | R: 5'-AAAGATGATTGGGGAGGGAG-3' |
| JHDM1D | F: 5'-TGCTGTTGACATTGACCTGT-3' |
|  | R: 5'-ATTTCATCGGCACTTGGGAA-3' |
| SOX2 | F:5’-CAGGAGTTGTCAAGGCAGAGA-3’ |
|  | R:5’-CCGCCGCCGATGATTGTTA-3’ |
| ΖΕΒ1 | F:5’-CCCAGTTACCCACAATCGTG-3’ |
|  | R:5’-AGGGCTGACCGTAGTTGAGTA-3’ |
| EZH2 | F:5’-TCCTTTTCATGCAACACCCA-3’ |
|  | R:5’-TTTCAGTCCCTGCTTCCCTA-3' |

| FOXM1 promoter | F: 5'-CGGTCTATTATATCCGAAGG-3' |
| --- | --- |
|  | R: 5'-CTTTTCAAAGCTCGGCTTTA-3' |
| TOP2A promoter | F: 5'-CGCCTCCCTAACCTGATTGG-3' |
|  | R: 5'-CTGACTCGCTCTCACCGTCT-3' |
| p21 promoter | F: 5'-CCAGAAAGGGGGCTCATTCTAA-3' |
|  | R: 5'-CGTTGGTGCGCTGGACACA-3' |
| CCNB1 promoter | 5′-GGAGCAGTGCGGGGTTTA-3′ |
|  | 5′-CGACCAGCCAAGGACCTACA-3′ |
